# Regioisomeric Control of Charge Transport Properties in Fluoranthene-Fused 12-Ring Heteroarenes

**DOI:** 10.1021/jacsau.5c00503

**Published:** 2025-06-18

**Authors:** Xinyu Yu, Chu-Yen Tsai, Yu-Wei Chu, Chu-Chen Chueh, Zhong'an Li

**Affiliations:** † Hubei Key Laboratory of Material Chemistry and Service Failure, Key Laboratory for Material Chemistry of Energy Conversion and Storage, Ministry of Education, School of Chemistry and Chemical Engineering, 12403 Huazhong University of Science and Technology, Wuhan 430074, P. R. China; ‡ Department of Chemical Engineering, 33561 National Taiwan University, Taipei 10617, Taiwan

**Keywords:** Heteroarene, Regioisomerism, Organic semiconductor, Charge transport property

## Abstract

Heteroarene-based organic semiconductors (OSCs) have emerged as promising material candidates for large-area, flexible electronic and photonic devices due to their favorable π-conjugation systems and tunable optoelectronic properties. However, their development is still hindered by synthetic and design challenges, in particular, the limited access to highly polycyclic heteroarenes with tunable properties and the unexplored effects of topological isomerism. Here, we have successfully synthesized three regioisomeric thienoacenes (**BTFA4**-**6**) by fusing a dicyanofluoranthene unit, which have an identical 12-fused ring composition but different molecular topologies with [a] or [c]-fusion and syn or anti-CN substitution. We demonstrate that solution-processable **BTFA4–6** exhibits topology-dependent charge transport properties by fabricating organic field-effect transistors. **BTFA4** with [a]-fusion and syn-CN substitution and **BTFA6** with [c]-fusion and syn-CN substitution show only typical n-type semiconducting behavior, with electron mobilities of 3.91 × 10^–4^ and 1.58 × 10^–5^ cm^2^ V^–1^ s^–1^, respectively, while **BTFA5** with [a]-fusion and anti-CN substitution achieves electron-dominated ambipolar behavior, with an increase in electron mobility (3.45 × 10^–2^ cm^2^ V^–1^ s^–1^) by 2 orders of magnitude and a hole mobility of 4.31 × 10^–3^ cm^2^ V^–1^ s^–1^. Therefore, this work establishes that regioisomeric molecular engineering is a powerful tool for manipulating the charge transport properties of heteroarene-based OSCs.

## Graphical abstract

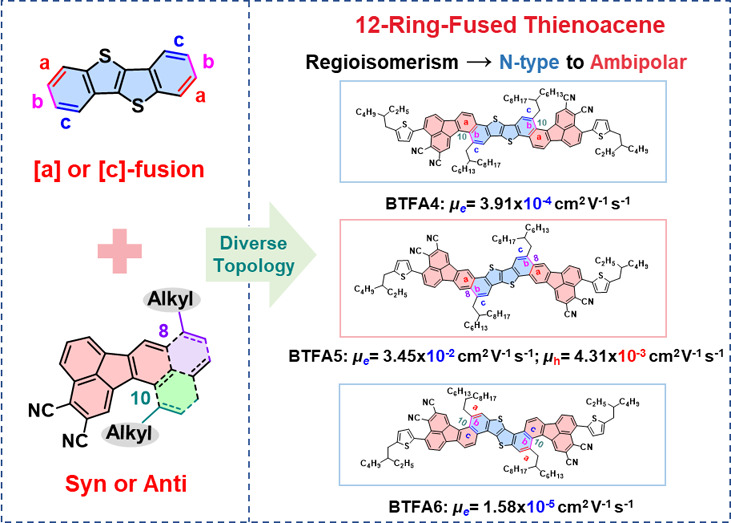

## 1 Introduction

Polycyclic aromatic hydrocarbons (PAHs) are key π-conjugated frameworks of organic semiconductors (OSCs) due to their attractive optical and electronic properties.^1–3^ The incorporation of heteroatoms (e.g., chalcogen and nitrogen atoms) into PAHs can delocalize electrons and thus improve their physicochemical stability.^4,5^ Furthermore, extended π-conjugated systems are particularly advantageous because their enhanced π–π interactions facilitate long-range molecular ordering and efficient charge transport.^6,7^ However, due to synthetic limitations and molecular design challenges, most of the reported heteroarenes are restricted to less than 10-fused rings, and very few of them can be developed into high-performance solution-processable OSCs.^8–11^

The molecular topology of heteroarenes, particularly the fusion sites and the orientation of the heteroatoms, has been shown to critically affect their material properties. These structural variations produce distinct regioisomers that dictate the molecular geometry and intramolecular electron distribution, thereby modulating intermolecular interactions and the solid-state packing.^12,13^ Such isomerism effect is fundamental to establishing the structure–property relationships of fused-ring OSCs.^14–16^ Although previous studies have highlighted the differences between angularly and linearly fused rings, the actual fused patterns of multiring-fused heteroarenes are much more intricate, due to the combination of both structures.^17,18^ Therefore, it is imperative to develop multiring-fused heteroarene-based OSCs with diverse molecular topologies.

Due to the harsh reaction conditions and limited building block diversity, current synthetic methods are still insufficient to create various regioisomeric heteroarenes.^19,20^ Thus far, most reported heteroarenes rely on heteroatom fusion and/or functional group substitution to tune the properties of material.^21–23^ For example, heteroarenes containing thiophene and pyrrole units are typically p-type OSCs but can be converted to n-type OSCs when containing pyrazine and imide groups.^24–27^ Fluoranthene, one of the representative PAHs, features a rigid and planarized structure with enhanced π–π interactions to facilitate intermolecular charge transport.^28–30^ Without special functionalization, its central cyclopenta ring imparts fluoranthene with distinctive electron-deficient characteristics, highlighting its potential in the design of π-conjugated materials.^31–33^ Our previous work pointed out that fluoranthene can be used in a highly efficient 2,3-functionalization strategy with highly electron-deficient groups (such as cyano and imide) through a facile Diels–Alder reaction, thereby obtaining a new class of fluoranthene-based OSCs with tunable charge transport properties.^34–36^ We also found that, due to their different reactivity, 2,3-substitued fluoranthene units can be selectively functionalized at the 4,9-positions, allowing the synthesis of regioregular dimers that can be used to investigate regioisomeric effects.^37^

Herein, as shown in Figure 1, through rational design of reactants and catalytic systems, we have incorporated a 2,3-dicyanofluoranthene unit into a linear 4-ring-fused thienoacene, and through a facile intramolecular alkyne-arene cycloisomerization reaction, three new 12-ring-fused heteroarene-based OSCs (**BTFA4**-**6**) with good solution processability have been constructed. Due to the changes in [a]- or [c]-fusion and syn or anti-CN orientations with respect to the alkyl chains of the thienoacene core, **BTFA4–6** has a diverse molecular topological structure, which affects the resulting photophysical, thermal, morphological, and electrical properties. Unlike the hole transport capabilities of reported heteroarene analogues,^41,44,45^ **BTFA5** exhibits electron-dominated ambipolar behavior, while **BTFA4** and **BTFA6** show typical n-type behavior. More encouragingly, the electron mobility of **BTFA5** (3.45 × 10^–2^ cm^2^ V^–1^ s^–1^) is much higher than that of **BTFA4** (3.91 × 10^–4^ cm^2^ V^–1^ s^–1^) and **BTFA6** (1.58 × 10^–5^ cm^2^ V^–1^ s^–1^). These results clearly indicate that regioisomerism is a key factor in determining the charge transport properties of heteroarene-based OSCs.

**Figure 1:**
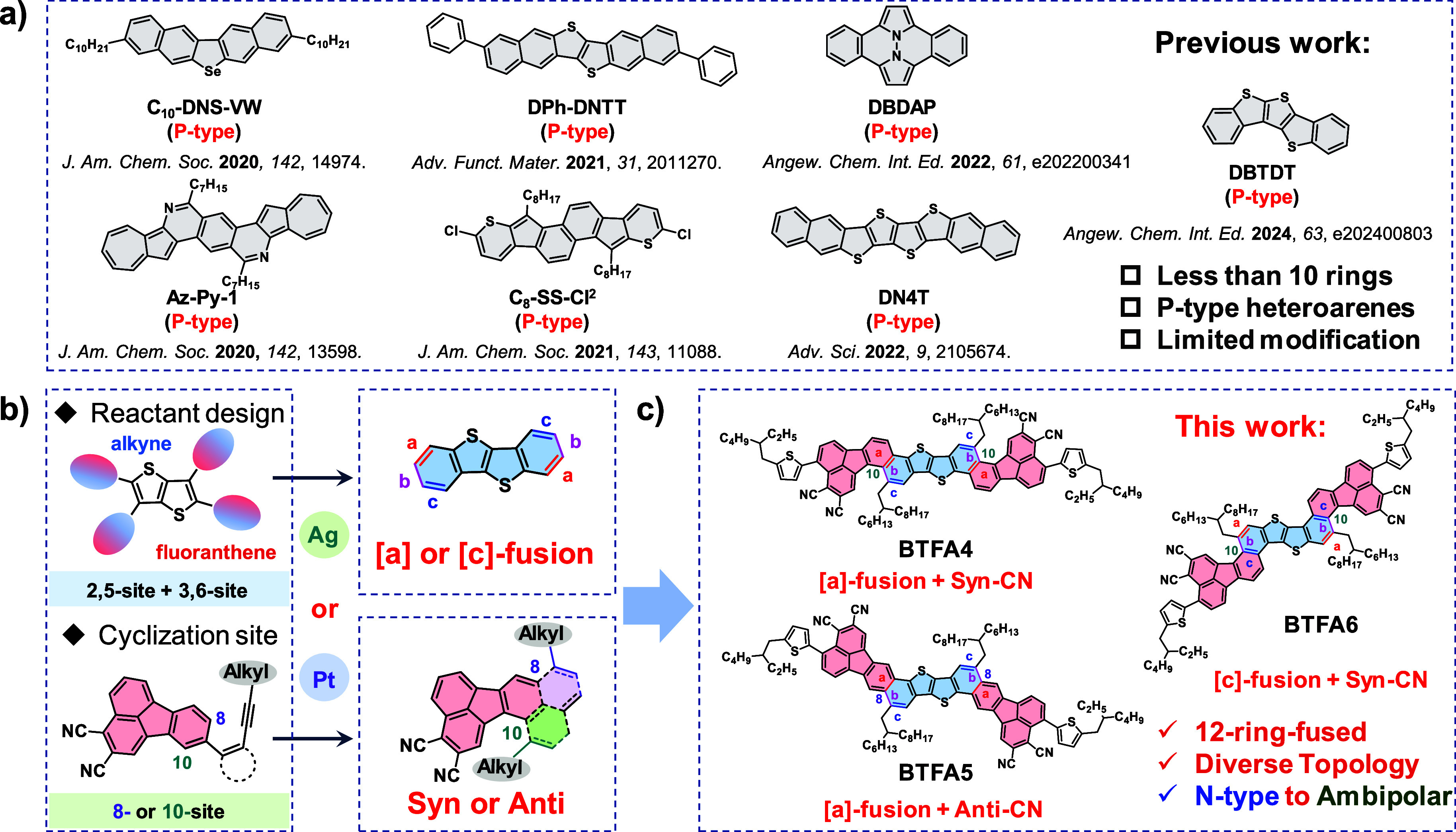
(a) The molecular structures of representative heteroarenes analogues;^22,38–43^ (b) schematic diagram of the design of 12-ring-fused heteroarenes based on 2,3-dicyanofluoranthene; (c) the molecular structures of **BTFA4–6** studied in this work.

## 2 Results and Discussion

To date, the rational design of strong electron-deficient building blocks with a compact and π-extended topological structure remains a formidable synthetic challenge, limiting the development of n-type OSCs.^46,47^ In our previous work, 2,3-substituted fluoranthene units have shown great promise in introducing electron-withdrawing groups, which can lower the lowest unoccupied molecular orbital (LUMO) energy level and improve electron-transport ability.^35,36,48^ Herein, as shown in Scheme 1, we have successfully synthesized a series of 12-ring-fused heteroarenes through a facile and well-defined synthetic route using 2,3-dicyanofluoranthene and thioacene as key building blocks. To ensure optimal processability, 2-ethylhexane and 2-hexyldecane chains were strategically introduced into the framework core and thiophene terminals, respectively.

**Scheme 1:**
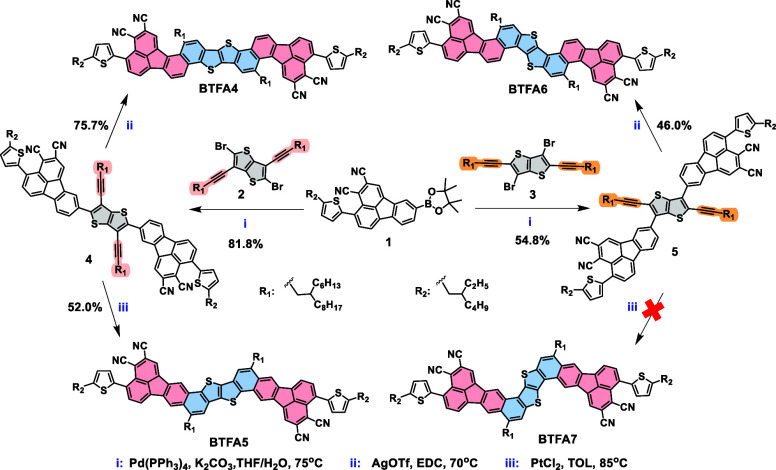
Synthetic Routes of **BTFA4-6**

First, a borate derivative of 2,3-dicyanofluoranthene (**1**) was synthesized to react with thieno­[3,2-*b*]­thiophene isomers having different substitution sites of bromine and alkynes (**2** and **3**) via the Suzuki reaction. The resulting key intermediates (**4** or **5**) then underwent a metal-catalyzed intramolecular alkyne–arene cycloisomerization reaction to obtain the desired 12-ring-fused heteroarenes with [a]-fusion or [c]-fusion. An interesting finding in this process is that different catalytic systems lead to changes in the reactive sites during annulation to obtain syn and anti-CN orientations relative to the alkyl chains. With silver trifluoromethanesulfonate (AgOTf) as a catalyst in 1,2-dichloroethane (EDC),^49^ the cycloisomerization reaction takes place at the 10-site of fluoranthene in intermediates **4** and **5** to yield **BTFA4** and **BTFA6**, respectively. On the other hand, using platinum dichloride (PtCl_2_) as a catalyst,^50^ the intermediate **4** can undergo cycloisomerization at the 8-site of fluoranthene to form **BTFA5**. However, the same reaction of the intermediate **5** failed, possibly caused by the different electron cloud densities of the active sites and the steric hindrance effect.^51^ Overall, the synthetic route integrating the design of isomeric intermediates and different catalytic systems is proven to be simple and efficient and can be used to obtain the 12-ring-fused regioisomers. All of the compounds have good solubility in common organic solvents. Synthetic details and characterization data are provided in the Supporting Information (SI).

As seen in Figure 2a, the ^1^H NMR spectra clearly confirmed the molecular structures of **BTFA4–6**. Moreover, the chemical shifts of the protons at the 1-site of fluoranthene (H^a^) and the range widths of the aromatic proton signals follow the order of **BTFA5** > **BTFA4** > **BTFA6**, thereby indicating that they possess distinct electron delocalization, and **BTFA5** has a more effective push–pull effect.^52,53^ The geometric and electronic properties of **BTFA4–6** were simulated using density functional theory (DFT) at the B3LYP/6–31G (d, p) level with Grimme’s D3BJ empirical dispersion correction. The long alkyl chains were replaced with methyl groups for structural simplification. Figure 2b shows the distinct molecular geometries of **BTFA4–6**, which dictate the molecular polarity: **BTFA4** (helical, μ ≈ 0 D), **BTFA5** (planar, μ = 2.50 D), and **BTFA6** (curved, μ = 6.45 D). This illustrates the regioisomeric effect on the charge asymmetry of OSCs, which affects their intermolecular interactions. While **BTFA4–6** almost have the same highest occupied molecular orbital (HOMO)/LUMO energy levels (Figure S1), **BTFA5** has an extended HOMO distribution and a slightly higher LUMO level, indicating enhanced π-conjugation and delocalization of electrons, which is confirmed by its wider range of electrostatic potential values (Figure 2c).

**Figure 2:**
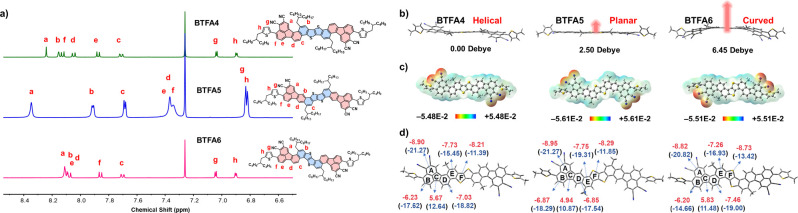
(a) ^1^H NMR spectra of **BTFA4–6** in CDCl_3_, (b) DFT-optimized geometry (side-view), (c) electrostatic potential distribution, and (d) NICS(0) (top)/NICS(1)_ZZ_ (bottom, parenthesized) values of **BTFA4–6**.

To further investigate the regioisomeric effects on aromaticity, nuclear independent chemical shift (NICS) values (Figure 2d) were also simulated using the B3LYP/6–31G­(d,p) level and gauge-including atomic orbital (GIAO) method.^9,38^ All conjugated rings in these heteroarenes exhibit negative NICS values, with the exception of cyclopenta ring **C**, whose positive NICS value indicates its antiaromatic character. Notably, the dicyano-substituted ring **A** shows the most negative NICS values, indicating optimal aromatic stabilization. Comparative analysis also reveals distinct aromaticity among the three regioisomers. The ring **C** in **BTFA5** shows the smallest positive NICS values (NICS(0)/NICS(1)_ZZ_: 4.94/10.87 ppm), suggesting weaker antiaromaticity relative to those in **BTFA4** and **BTFA6**. Moreover, the rings **B** and **E** in **BTFA5** exhibit the largest negative NICS values (NICS(0)/NICS(1)_ZZ_: −6.87/−18.29 and −7.75/−19.31 ppm, respectively), and the rings **D** and **F** in **BTFA6** show greater aromatic stabilization with NICS(0)/NICS(1)_ZZ_ of −7.46/−19.00 and −8.73/−13.42 ppm, respectively. It is obviously demonstrated that regioisomerism governs electron delocalization character throughout the π-framework, where specific fusion geometries dictate unique aromatic stabilization profiles via distinct conjugation pathways.

The optical, electrochemical, and thermal properties of **BTFA4–6** have been thoroughly characterized and are listed in Table 1. As shown in Figure 3a, **BTFA4** and **BTFA6** both exhibit similar absorption profiles, while **BTFA5** shows a red-shifted ICT peak at 510 nm and a higher absorption coefficient, further confirming that the planar molecular topology of **BTFA5** enhances the push–pull effect. This is also attributed to their different electronic structures, which are governed by conjugation and induction effects. As mentioned above, the significant downfield shift of H^a^ and the expanded electrostatic potential range collectively suggest a more delocalized π-electron system in **BTFA5**. When converted from the solution state to the film state, **BTFA6** experienced a more significant redshift of the absorption band than **BTFA4** (Figure 3b), indicating stronger aggregation, which could be due to dipole–dipole interactions caused by its more polar structure.^54,55^ Meanwhile, **BTFA5** exhibits a split ICT peak with a red-shift of 21 nm for the long-wavelength peak, which implies an obvious change in the ground state electronic structure after aggregation. As discussed above, compared with **BTFA4** and **BTFA6**, the planar structure of **BTFA5** will favor intermolecular π–π interactions, thereby enhancing the cofacial stacking of molecules.^56–58^

**Figure 3:**
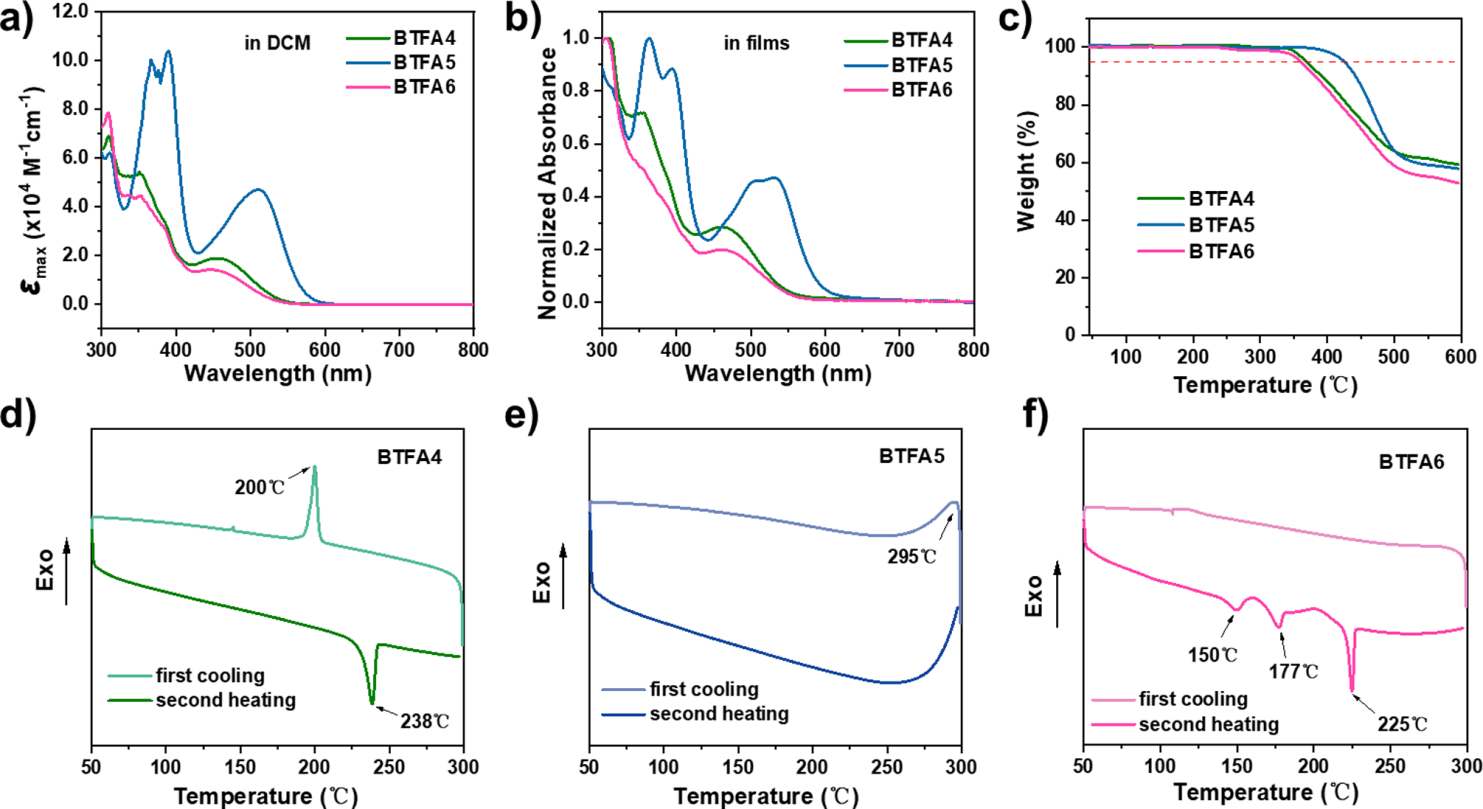
Absorption spectra of **BTFA4–6** (a) in CH_2_Cl_2_ solutions and (b) in films. (c) TGA curves of **BTFA4–6** measured in N_2_ at a heating rate of 10 °C/min. DSC curves of (d) **BTFA4**, (e) **BTFA5**, and (f) **BTFA6** measured in N_2_ at a heating rate of 10 °C/min.

**Table 1:** Optical, Electrochemical, and Thermal Properties of **BTFA4-6**

materials	λ_max,sol_ ^a^ (nm)	λ_max,fil_ ^a^ (nm)	λ_onset,sol_ ^b^ (nm)	λ_onset,fil_ ^b^ (nm)	*E*_g_ ^c^ (eV)	*E*_HOMO_ ^d^ (eV)	*E*_LUMO_ ^e^ (eV)	*T*_d_ ^f^ (°C)	*T*_m_ ^g^ (°C)
**BTFA4**	455	461	537	545	2.31	–5.75	–3.44	368	238
**BTFA5**	510	531,510	571	588	2.17	–5.62	–3.45	425	/
**BTFA6**	448	461	534	554	2.32	–5.78	–3.46	360	225

a The maximum wavelength of the ICT absorption band in solutions and thin films.

b The long wavelength tail of solutions and thin films.

c Optical bandgap calculated from long-wavelength tail of solutions.

d Calculated by the equation of *E*_HOMO_ = *E*_LUMO_ − *E*_g_.

e Measured from electrochemistry experiments in solutions using ferrocene as the reference.

f The 5% weight loss temperature detected by the TGA analysis.

g The melting temperature detected by the DSC analysis.

The energy levels of **BTFA4–6** were determined using cyclic voltammetry (CV) with Fc/Fc^+^ as the reference. As shown in Figure S2, they all show similar LUMO levels around −3.45 eV. Interestingly, only **BTFA5** was able to produce a meaningful CV curve during the positive sweep, with an estimated HOMO level of –5.51 eV. Such a phenomenon indicates electron transfer accompanied by the formation of a cation, which may confer the ambipolar property of **BTFA5** due to its more delocalized electron distribution. We further calculated the HOMO energy levels of **BTFA4** and **BTFA6** based on the equation *E*_HOMO_ = *E*_LUMO_ – *E*_g_, which are −5.75 and −5.78 eV, respectively. Thermogravimetric analysis (TGA, Figure 3c) under a nitrogen atmosphere revealed that the heteroarenes have excellent thermal stability, with decomposition temperatures following the trend: **BTFA5** (425 °C) > **BTFA4** (368 °C) > **BTFA6** (360 °C). Furthermore, the differential scanning calorimetry (DSC) curves in Figure 3d–f show significantly different thermal behaviors. During the first cooling process, **BTFA5** exhibited a broad exothermic peak at 295 °C, indicating its weak crystallinity. **BTFA4** displayed a sharp melting peak with a melting point (*T*_m_) of 238 °C, while **BTFA6** showed two phase-transition peaks prior to the melting peak at 225 °C. Therefore, the multiple endothermic processes observed during heating of **BTFA6** indicate its stronger crystallinity and multiple stacking patterns. As a result, these regioisomers exhibit distinct thermal stabilities, which is probably mainly due to their distinct aromaticity and molecular packing behavior resulting from their distinctive molecular topologies.

To investigate the regioisomeric effects on the film morphology, we used atomic force microscopy (AFM) to characterize **BTFA4–6** films prepared by a typical spin coating process. As shown in Figure 4a,b, these heteroarenes exhibit different film morphologies. Specifically, the **BTFA4** film displays cracks, the **BTFA5** film shows nanograins, and the **BTFA6** film displays wires. The root-mean-square (RMS) roughness values of **BTFA4–6** films are determined to be 1.77, 2.63, and 1.33 nm, respectively, which is consistent with the planarity and conjugation affected by the topology. Although the surfaces of these films are relatively smooth, with an RMS roughness value of less than 3 nm, the texture differences are obvious from the optical images (Figure 4c). Compared with the **BTFA4** film with larger grain boundaries, the continuous film morphology and larger aggregates of **BTFA5** may enhance charge mobility. Although **BTFA6** maintains a single-crystal-like order, its discontinuous wire-like morphology may lead to the disorder of electron transport pathways.

**Figure 4:**
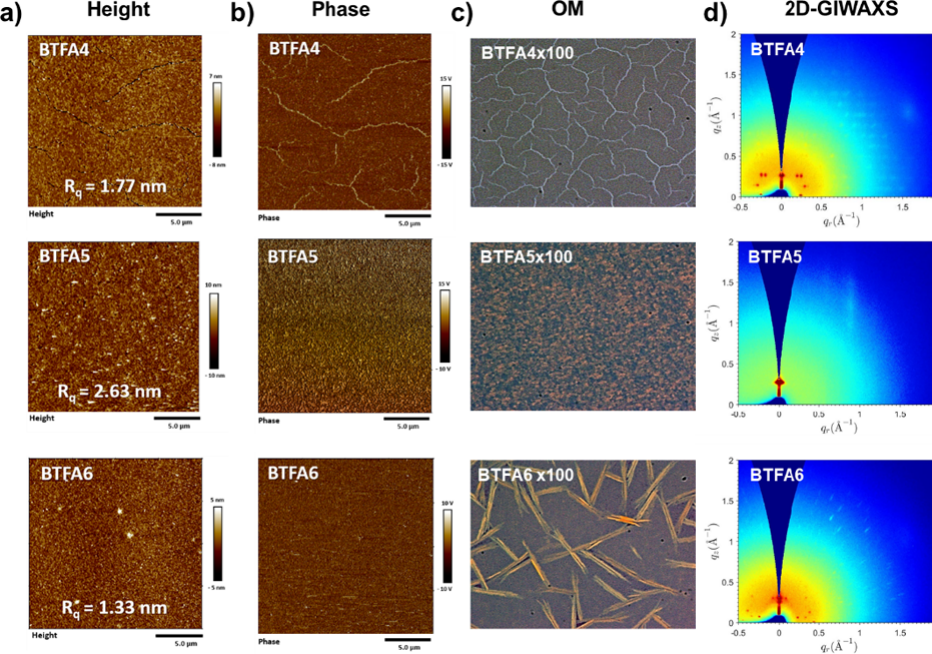
(a) AFM height images, (b) phase images, (c) optical images, and (d) 2D-GIWAXS patterns of the films of **BTFA4–6**.

Grazing incidence wide-angle X-ray scattering (GIWAXS) was further performed to precisely characterize the crystallographic properties and stacking structures of **BTFA4–6** films, as depicted in Figure 4d. The crystallographic plane, stacking distance, and crystallographic coherence length (CCL) were extracted from the corresponding one-dimensional (1D) profiles in the out-of-plane (OOP) direction (Figure S3). In the two-dimensional (2D) GIWAXS patterns, **BTFA5** mainly has a (001) diffraction peaks. In contrast, **BTFA4** and **BTFA6** show numerous diffraction peaks, indicating multiple stacking behavior, among which **BTFA4** shows a more ordered arrangement than **BTFA6**. The results demonstrate that an increase in the π-conjugation of the fused rings leads to a decrease in crystallinity, but the continuity of the film improves. This is related to differences in intermolecular interactions derived from different molecular topologies. We infer that **BTFA5** ([a]-fusion and anti-CN) has a more planar geometry and a better delocalized electron distribution, showing stronger π–π interactions.

We then evaluated the charge transport properties of **BTFA4–6** by fabricating organic field-effect transistors (OFETs) with the bottom-gate and top-contact (BGTC) structure. Specifically, a 5 mg/mL chloroform solution of **BTFA4–6** was spin-coated on a highly n-doped silicon (100) wafer with a 300 nm thick SiO_2_ gate dielectric treated with octadecyltrichlorosilane (ODTS). The detailed fabrication process is described in the SI. Under optimized conditions, as shown in Figure 5, all **BTFA4–6**-based OFETs exhibit typical transfer characteristics, with the related data summarized in Table 2. Figure S4 presents the output characteristics of **BTFA5**, which display a clear and typical transistor behavior. The output curves for **BTFA4** and **BTFA6** were not shown due to their poor performance and lack of well-defined electrical characteristics. This may be attributed to low mobility and unfavorable molecular stacking.

**Figure 5:**
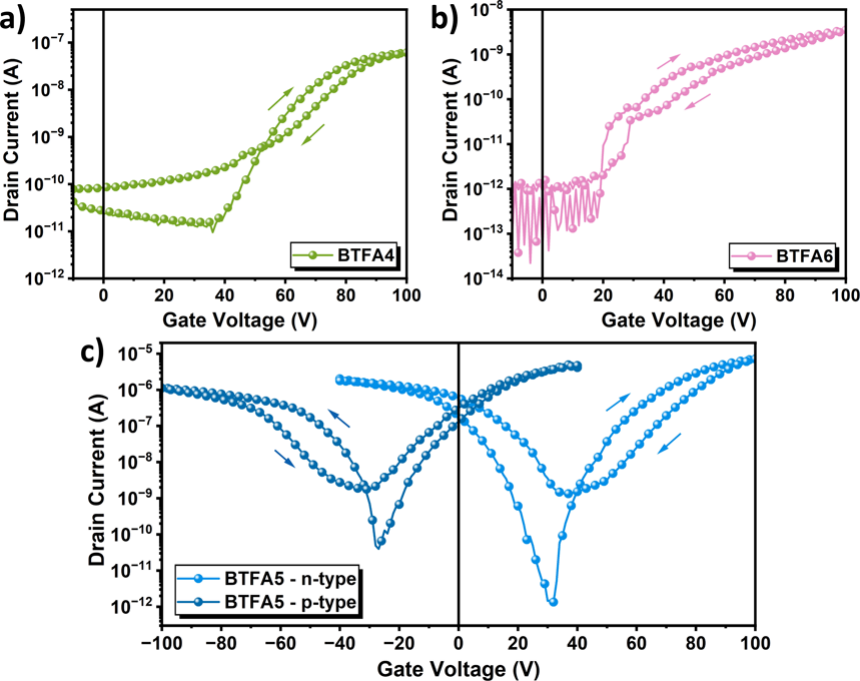
n-Type transfer characteristics of the OFET devices based on (a) **BTFA4** and (b) **BTFA6**. (c) Ambipolar transfer characteristics of the OFET device based on the **BTFA5**.

**Table 2:** OFET Characteristics in Different Experimental Conditions^a^

materials	RPM	annealing temp. (°C)	annealing time (min)	type	μ_average_ ^a^ (cm^2^ V^–1^ s^–1^)	average on/off	average *V*_t,sat_ (V)
**BTFA4**	1000	180	30	N	3.91 × 10^–4^ ± 6.91 × 10^–5^	10^6^–10^7^	50.21
**BTFA5**	2000	150	30	N	3.45 × 10^–2^ ± 8.96 × 10^–3^	10^4^–10^6^	54.88
	2000	150	30	P	4.31 × 10^–3^ ± 1.70 × 10^–3^	10^4^–10^5^	–43.98
**BTFA6**	2000	150	30	N	1.58 × 10^–5^ ± 5.11 × 10^–6^	10^5^–10^6^	43.59

a The mobility values were averaged from at least 25 devices made from 4 different batches.

Both **BTFA4** and **BTFA6** exhibited n-type electron transport behavior due to their fusion with the highly electron-deficient 2,3-dicyanofluoranthene units. The helical **BTFA4** has a superior electron mobility (μ_e_ = 3.91× 10^–4^ cm^2^ V^–1^ s^–1^), outperforming that of the curved analogue **BTFA6** (μ_e_ = 1.58 × 10^–5^ cm^2^ V^–1^ s^–1^). This result shows that a higher degree of crystallinity does not necessarily guarantee better charge transport properties, but a well-aligned molecular stacking structure and a more effective electron delocalization may be more conducive to electron transport.^59–62^ In contrast, **BTFA5** has a significantly high μ_e_ of 3.45 × 10^–2^ cm^2^ V^–1^ s^–1^ compared to those of **BTFA4** (3.91 × 10^–4^ cm^2^ V^–1^ s^–1^) and **BTFA6** (1.58 × 10^–5^ cm^2^ V^–1^ s^–1^), and its *V*_t_sat_ value is 54.88 V. Its hole mobility is also measured as 4.31 × 10^–3^ cm^2^ V^–1^ s^–1^ with a *V*_t_sat_ of −43.98 V. As a result, the planar **BTFA5** exhibits a superior electron transport property and an interesting ambipolar charge transport capability. These results strongly suggest that regioisomerism affects the molecular geometry, electron distribution, and aggregation behavior, which, in turn, affects the charge transport properties of OSCs. It is noteworthy that these 12-ring-fused heteroarene-based OSCs achieve remarkable charge transport properties through direct solution processing without any solvent additives (e.g., 1,8-diiodooctane, chloronaphthalene, etc.). These commonly used additives are reported to improve solubility and film quality but often affect device reliability and operational stability due to residual contamination.^63–65^

Therefore, the above results strongly indicate that geometric and electronic structures are two sides of the same coin due to the regioisomeric effect. It is evident that both structures play crucial roles in determining the intermolecular interactions and molecular stacking behavior. The planar molecular topology of **BTFA5** and its more effective charge delocalization enable it to exhibit adequate π–π and dipole–dipole intermolecular interactions in the solid state, resulting in a continuous film morphology with controlled aggregations. Consequently, **BTFA5** exhibits good ambipolar charge transport properties.

## 3 Conclusions

In summary, we have established an efficient synthetic strategy for the regioisomeric 12-ring-fused heteroarenes, which are fused from thioacene and 2,3-dicyanofluoranthene units at different sites to form [a] or [c] fusions and syn- or anti-CN orientations. We have demonstrated that the molecular topology of regioisomers has a significant effect on the geometry, electron distribution, and aggregation behavior of heteroarenes, which in turn play a crucial role in determining their material properties. Compared to **BTFA4** ([a]-fusion and syn-CN) and **BTFA6** ([c]-fusion and syn-CN), **BTFA5** ([a]-fusion and anti-CN) has more red-shifted absorption and higher thermal stability. It is noteworthy that this even leads to a transition in the charge transport properties from n-type semiconducting behavior (**BTFA4** and **BTFA6**) to ambipolar behavior (**BTFA5**). In particular, **BTFA5** achieves an electron mobility of 3.45 × 10^–2^ cm^2^ V^–1^ s^–1^, which is much higher than those of **BTFA4** (3.91 × 10^–4^ cm^2^ V^–1^ s^–1^) and **BTFA6** (1.58 × 10^–5^ cm^2^ V^–1^ s^–1^), while the hole mobility reaches 4.31 × 10^–3^ cm^2^ V^–1^ s^–1^, showing balanced ambipolar characteristics. We believe that this work not only provides valuable insights into the synthesis of solution-processable polycyclic heteroarene-based OSCs but also elucidates the impact of regioisomeric effects on charge transport properties.

## Supplementary Material



## Notes

Detailed description of the experimental section, supporting tables, supporting figures, and supporting references (PDF)

## Notes

Dedicated to Professor Alex Jen on the Occasion of His 70th Birthday.
