# Supplementary material for: Regioisomeric Control of Charge Transport Properties in Fluoranthene-Fused 12-Ring Heteroarenes
Source: JACS Au. 2025 Jun 18;5(7):3483–90. doi: 10.1021/jacsau.5c00503 (PMC12308440; doi:10.1021/jacsau.5c00503)
Supplement: Supplementary file 1 [file au5c00503_si_001.pdf]

Supporting Information

Regioisomeric Control of Charge Transport Properties in Fluoranthene-Fused 12-Ring Heteroarenes

Xinyu Yu,[a] *Chu-Yen Tsai,*[b] *Yu-Wei Chu,*[b] *Chu-Chen Chueh,*[b,*] *and Zhong'an Li*[a,*]

[a] Key Laboratory for Material Chemistry of Energy Conversion and Storage, Ministry of Education, Hubei Key Laboratory of Material Chemistry and Service Failure, School of Chemistry and Chemical Engineering, Huazhong University of Science and Technology, Wuhan, 430074, China.

E-mail: lizha@hust.edu.cn

[b] Department of Chemical Engineering, National Taiwan University, Taipei, 10617, Taiwan.

E-mail: cchueh@ntu.edu.tw

Table of Contents

1. Experimental Procedures S3
2. Supporting Figures S11
3. Reference S23

1. Experimental Procedures

Materials and instruments

1,4-dioxane, toluene (TOL), tetrahydrofuran (THF) was dried and distilled from sodium under an atmosphere of dry nitrogen before use. Compound **1a**,[1] **3a**,[2] tributyl(5-(2-ethylhexyl)thiophen-2-yl)stannane,[3] and 7-(prop-2-yn-1-yl)pentadecane[4] was synthesized as previously reported. Other reagents were purchased from Sigma-aldrich, TCI, Energy Chemical and general sources and all commercial materials were used as received without re-purification unless otherwise stated.

^{1}H NMR and ^{13}C NMR spectra were recorded on Bruker Avance 400 MHz or 600 MHz. Mass spectrometry was performed using a Applied Biosystems 4700 Proteomics Analyzer MALDI-TOF/TOF. UV-Vis-NIR absorption spectra were performed on Perkin Elmer Lamda 750s spectrometers. CV was carried out in degassed CH_2Cl_2 (DCM) with $BuN^+PF_6^-$ (0.1 mol/L) as electrolyte on a CHI650E electrochemical analyser (CH Instruments, Inc., China). A three-electrode system was used with Pt disc, Pt wire and Ag/AgCl as working, counter and reference electrodes, respectively. TGA and DSC were investigated by TA instruments TGA Q500 and Perkin Elmer Pyris I, respectively, under a nitrogen atmosphere with a heating rate of 10 ℃/min.

The surface roughness and morphology of the small molecule films were characterized in ambient conditions using a 3D controller atomic force microscope (AFM, Digital Instrument) in tapping mode. Scans were performed over a 5 μm × 5 μm area to evaluate surface roughness, grain size, and film continuity. Optical microscopy (OM) images were obtained under ambient conditions using a standard bright-field microscope to visualize large-area film morphology. Grazing-incidence wide-angle X-ray scattering (GIWAXS) measurements were performed at the 13A1 beamline of the National Synchrotron Radiation Researcher Center (NSRRC) in Taiwan. X-rays with a wavelength of 1.02739Å and an incident angle of 0.12° were used to study the crystal structure, and the calculation of the crystallographic coherence length (CCL) is described in detail below. Single-crystal X-ray diffraction

data were collected using a Rigaku XtaLAB PRO MM007HF instrument and further analysed using Mercury software to understand the crystal structure in conjunction with the GIWAX results. FET characteristics were measured in a nitrogen-filled glovebox using a Keithley 4200-SCS semiconductor parameter analyzer (Keithley Instruments Inc.) and the detailed calculation of mobility is described in detail below. The small molecule films used to fabricate the FETs and for the UV, AFM, and GIWAXS analyses were prepared by the same spin-coating method.

Device fabrication and characterizations

We fabricated a bottom gate/top contact (BG/TC) FET for the small molecules under study. The substrate was a highly n-doped silicon (100) wafer, with a 300 nm thick SiO_2 gate dielectric and a capacitance of about 10 nF/cm^2. An octadecyltrichlorosilane (ODTS) self-assembled monolayer was deposited on the SiO_2 surface to improve the surface roughness and promote the formation of the crystal structure of the small molecules under study. The active layers were prepared by spin-coating a 5 mg/mL chloroform solution of each BTFA compound at either 1000 rpm (for **BTFA4**) or 2000 rpm (for **BTFA5** and **BTFA6**) for 60 s. The films were then annealed at their optimized temperatures respectively: 180 °C for **BTFA4**, and 150 °C for **BTFA5** and **BTFA6**, each for 30 minutes. Finally, 60 nm of Au was deposited via a regular shadow mask to define the top contact source and drain electrodes with a channel length (L) of 100 μm and a channel width (W) of 2000 μm.

Calculation of electron mobility

The mobility value is calculated from the transfer curve at the saturated regime according to the following formula:

$$I_{DS} = \left(\frac{W}{2L}\right)C\mu_{sat}\,(V_{GS} - V_{th})^2$$

where I_{DS} is the drain current, W and L are the channel width and channel length, respectively, C is the areal capacitance of the dielectric layer, μ_{sat} is the mobility in the saturation region, and V_{GS} and V_{th} are the gate voltage and threshold voltage, respectively. The μ_{sat} value of the device can be determined from the relationship between the square root values of I_d and V_{GS}.

Calculation of crystallographic coherence length (CCL)

The crystallographic coherence length (CCL) is a parameter for analyzing crystallite size and is calculated as follows:

$$\mathrm{CCL} = \frac{2K}{\Delta_q}$$

where CCL is the coherence length, K is a dimensionless shape factor, which we define as 0.9, and Δ_q is the full width at half-maximum of the diffraction peak.

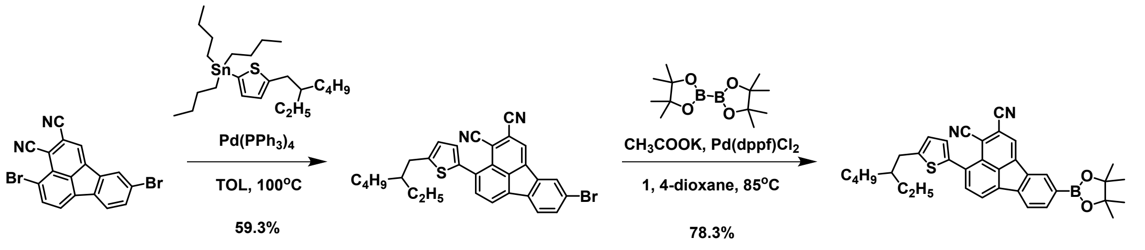

Synthesis of 1

Compound **1a** (1.00 g, 2.44 mmol), tributyl(5-(2-ethylhexyl)thiophen-2-yl)stannane (1.24 g, 2.56 mmol) and $Pd(PPh_3)_4$ (0.14 g, 0.12 mmol) were dissolved in 20 mL of dry TOL under N_2. Then the mixture was heated to 100 °C and stirred for 24 h. After the mixture cooled, it was diluted with DCM, washed with water and extracted with DCM. The combined organic layer was dried with anhydrous Na_2SO_4. After concentration using a rotary evaporator, the crude product was purified through column

chromatography (SiO_2, petroleum ether/DCM, 1.5/1, V/V) to afford **1b** (0.76 g, 59.3%) as an orange solid. ^{1}H NMR (600 MHz, Chloroform-*d*) δ 8.13 (s, 1H, ArH), 8.06 (d, *J* = 1.7 Hz, 1H, ArH), 8.02 (d, *J* = 7.2 Hz, 1H, ArH), 7.81 (d, *J* = 7.2 Hz, 1H, ArH), 7.77 (d, *J* = 8.1 Hz, 1H, ArH), 7.65 (dd, *J* = 8.1, 1.8 Hz, 1H, ArH), 7.01 (d, *J* = 3.5 Hz, 1H, ArH), 6.87 (d, *J* = 3.5 Hz, 1H, ArH), 2.86 (d, *J* = 6.7 Hz, 2H, $-CH_2-$), 1.70-1.60 (m, 1H, -CH-), 1.45-1.32 (m, 8H, $-CH_2-$), 0.93 (t, *J* = 7.6 Hz, 6H, $-CH_3$).

Compound **1b** (0.72 g, 1.37 mmol), 4,4,4',4',5,5,5',5'-octamethyl-2,2'-bi(1,3,2-dioxaborolane) (0.52 g, 2.06 mmol), CH_3COOK (0.27 g, 2.74 mmol) and Pd(dppf)Cl_2 (0.10 g, 0.14 mmol) were dissolved in 15 mL of dry 1, 4-dioxane under N_2. The mixture was heated to 85 °C for 18 h. Then the mixture was cooled to room temperature, and drop added into 150 mL of methanol. An orange solution was collected by filtration and concentrated by a rotary evaporator. The crude product was purified by recrystallization (petroleum ether) to obtain **1** as a reddish orange solid (0.61 g, 78.3%). ^{1}H NMR (600 MHz, Chloroform-*d*) δ 8.36 (s, 1H, ArH), 8.16 (s, 1H, ArH), 8.03 (d, *J* = 7.2 Hz, 1H, ArH), 7.96 (d, *J* = 7.5 Hz, 1H, ArH), 7.90 (d, *J* = 7.5 Hz, 1H, ArH), 7.80 (d, *J* = 7.1 Hz, 1H, ArH), 7.01 (d, *J* = 3.4 Hz, 1H, ArH), 6.87 (d, *J* = 3.5 Hz, 1H, ArH), 2.92-2.80 (m, 2H, $-CH_2-$), 1.70-1.62 (m, 1H, -CH-), 1.50-1.22 (m, 20H, $-CH_2-$, $-CH_3$), 0.96-0.88 (m, 6H, $-CH_3$).

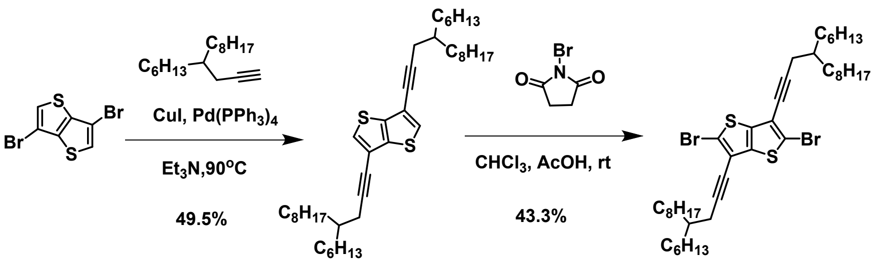

2a 2b 2

Synthesis of 2

Compound **2a** (0.70 g, 2.35 mmol), 7-(prop-2-yn-1-yl)pentadecane (2.35 g, 9.40 mmol), CuI (0.02 g, 0.12 mmol) and Pd(PPh_3)$_4$ (0.14 g, 0.12 mmol) were dissolved in 15 mL of degassed Et_3N under N_2. The mixture was heated to 90 °C and stirred for 36 h. After the mixture cooled, it was

concentrated by a rotary evaporator and then diluted with petroleum ether (PE). The solution was washed with water and dried with anhydrous Na_2SO_4. After concentration using a rotary evaporator, the crude product was purified through column chromatography (SiO_2, PE) to obtain **2b** as a yellow liquid (0.74 g, 49.5%). ^{1}H NMR (600 MHz, Chloroform-*d*) δ 7.37 (s, 2H, ArH), 2.42 (d, *J* = 5.8 Hz, 4H, -CH_2-), 1.63-1.58 (m, 2H, -CH-), 1.44-1.26 (m, 48H, -CH_2-), 0.90-0.85 (m, 12H, -CH_3).

Compound **2b** (0.50 g, 0.78 mmol) was dissolved in 10 mL of $CHCl_3$/AcOH (1/1, V/V) and put into the ice bath in dark. Then the 1-bromopyrrolidine-2,5-dione (0.31 g, 1.73 mmol) was added in three batches within 0.5 h. The mixture was stirred for 12 h, then washed with water and extracted with PE. The combined organic layer was dried with anhydrous Na_2SO_4. After concentration using a rotary evaporator, the crude product was purified through column chromatography (SiO_2, PE) to afford **2** (0.27 mg, 43.3%) as a brown liquid. ^{1}H NMR (600 MHz, Chloroform-*d*) δ 2.46 (d, *J* = 5.7 Hz, 4H, -CH_2-), 1.68-1.57 (m, 2H, -CH-), 1.48-1.26 (m, 48H, -CH_2-), 0.90-0.86 (m, *J* = 6.9, 5.7 Hz, 12H, -CH_3).

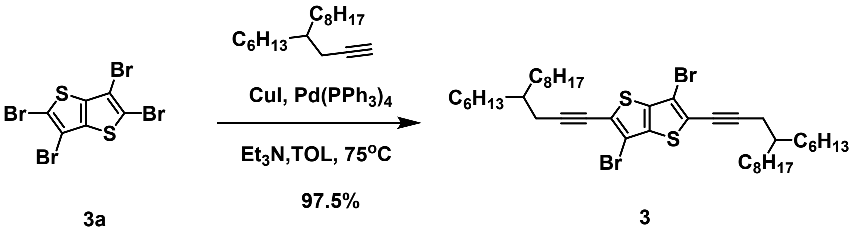

3a
3

Synthesis of 3

Compound **3a** (0.60 g, 1.32 mmol), 7-(prop-2-yn-1-yl) pentadecane (0.66 g, 2.63 mmol), CuI (0.03 g, 0.16 mmol) and $Pd(PPh_3)_4$ (0.06 g, 0.08 mmol) were dissolved in 15 mL of degassed Et_3N/TOL (2/1, V/V) under N_2. The mixture was heated to 75 °C and stirred for 36 h. After the mixture cooled, it was concentrated by a rotary evaporator and then diluted with PE. The solution was washed with water and dried with anhydrous Na_2SO_4. After concentration using a rotary evaporator, the crude product was purified through column chromatography (SiO_2, PE) to obtain **3** as a brown liquid (1.02 g, 97.5%). ^{1}H NMR (400 MHz, Chloroform-*d*) δ 2.49 (d, *J* = 5.8 Hz, 4H, -CH_2-), 1.66-1.61 (m, 2H, -

CH-), 1.43-1.26 (m, 48H, $-CH_2-$), 0.91-0.85 (m, 12H, $-CH_3$).

Synthesis of 4

A mixture of compound **2** (285 mg, 0.32 mmol), compound **1** (387 mg, 0.74 mmol), $Pd(PPh_3)_4$ (37 mg, 0.03 mmol), K_2CO_3 (177 mg, 1.28 mmol) and degassed THF/H_2O (10 mL, 2/1, V/V) was heated to 75 °C under N_2 for 24 h. After cooling to room temperature, the mixture was washed with water and extracted with DCM. The combined organic layer was dried with anhydrous Na_2SO_4. After concentration using a rotary evaporator, the crude product was purified through column chromatography (SiO_2, PE /DCM, 1/1, V/V) to afford **4** (400 mg, 81.8%) as a red solid. ^1H NMR (600 MHz, Chloroform-*d*) δ 8.40 (s, 2H, ArH), 8.11-8.07 (m, 4H, ArH), 7.96 (d, J = 7.1 Hz, 2H, ArH), 7.89 (d, J = 7.9 Hz, 2H, ArH), 7.77 (d, J = 7.1 Hz, 2H, ArH), 6.99 (d, J = 3.2 Hz, 2H, ArH), 6.86 (d, J = 3.2 Hz, 2H, ArH), 2.90-2.82 (m, 4H, $-CH_2-$), 2.62 (d, J = 5.7 Hz, 4H, $-CH_2-$), 1.75-1.63 (m, 4H, -CH-), 1.50-1.16 (m, 64H, $-CH_2-$), 0.97-0.90 (m, 12H, $-CH_3$), 0.85-0.78 (m, 12H, $-CH_3$).

Synthesis of 5

A mixture of compound **3** (300 mg, 0.34 mmol), compound **1** (408 mg, 0.78 mmol), $Pd(PPh_3)_4$ (39 mg, 0.03 mmol), K_2CO_3 (187 mg, 1.35 mmol) and degassed THF/H_2O (10 mL, 2/1, V/V) was heated to 75 °C under N_2 for 24 h. After cooling to room temperature, the mixture was washed with water and extracted with DCM. The combined organic layer was dried with anhydrous Na_2SO_4. After concentration using a rotary evaporator, the crude product was purified through column chromatography (SiO_2, PE /DCM, 1/1, V/V) to afford **5** (282 mg, 54.8%) as a red solid. ^1H NMR (400 MHz, Chloroform-*d*) δ 8.46-8.40 (m, 2H, ArH), 8.16 (s, 2H, ArH), 8.06-8.01 (m, 4H, ArH), 7.98 (d, J = 7.8 Hz, 2H, ArH), 7.82 (d, J = 7.1 Hz, 2H, ArH), 7.02 (d, J = 3.4 Hz, 2H, ArH), 6.88 (d, J = 3.4 Hz, 2H, ArH), 2.88 (d, J = 6.3 Hz, 4H, $-CH_2-$), 2.52 (d, J = 5.7 Hz, 4H, $-CH_2-$), 1.73-1.57 (m, 4H, -CH-), 1.46-1.12 (m, 64H, $-CH_2-$), 0.99-.90 (m, 12H, $-CH_3$), 0.84-0.76 (m, 12H, $-CH_3$).

Synthesis of BTFA4

Compound **4** (100 mg, 66 μmol) and AgOTf (5 mg, 20 μmol) were dissolved in 5 mL of dry 1,2-dichloroethane under N_2. The mixture was heated to 70 °C and stirred for 72 h. After cooling to room temperature, it was concentrated using a rotary evaporator and purified through column chromatography (SiO_2, PE /DCM, 1/1.5, V/V) to afford **BTFA4** (46 mg, 46.0%) as an orange solid. ^{1}H NMR (400 MHz, Chloroform-*d*) δ 8.24 (s, 2H, ArH), 8.15 (s, 2H, ArH), 8.13 (d, *J* = 7.2 Hz, 2H, ArH), 8.05 (d, *J* = 7.7 Hz, 2H, ArH), 7.87 (d, *J* = 7.2 Hz, 2H, ArH), 7.71 (dd, *J* = 7.8, 1.6 Hz, 2H, ArH), 7.04 (d, *J* = 3.4 Hz, 2H, ArH), 6.90 (d, *J* = 3.5 Hz, 2H, ArH), 2.88 (d, *J* = 6.6 Hz, 4H, $-CH_2-$), 2.58-2.46 (m, 4H, $-CH_2-$), 1.72-1.63 (m, 2H, -CH-), 1.53-1.33 (m, 18H, -CH-, $-CH_2-$), 1.22-0.83 (m, 66H, $-CH_2-$, $-CH_3$), 0.75 (t, *J* = 7.1 Hz, 6H, $-CH_3$). ^{13}C NMR (151 MHz, Chloroform-*d*) δ 196.10, 151.58, 147.85, 141.06, 140.17, 137.12, 136.61, 136.51, 135.13, 134.68, 134.35, 134.18, 133.85, 132.16, 130.59, 130.48, 128.70, 125.70, 124.86, 124.08, 122.21, 122.00, 120.34, 116.51, 114.32, 113.92, 41.53, 38.98, 37.03, 34.05, 33.32, 32.27, 31.85, 31.74, 29.95, 29.62, 29.49, 29.27, 28.92, 28.32, 26.46, 26.44, 25.35, 23.01, 22.64, 22.61, 14.20, 14.12, 14.08, 10.83. MS: $[M\bullet 2H_2O+H]^+$ calcd for $C_{102}H_{121}N_4O_2S_4^+$, 1562.8401; found, 1562.8099.

Synthesis of BTFA5

Compound **4** (100 mg, 66 μmol) and $PtCl_2$ (7 mg, 26 μmol) were dissolved in 5 mL of dry TOL under N_2. The mixture was heated to 85 °C and stirred for 24 h. After cooling to room temperature, it was concentrated using a rotary evaporator and purified through column chromatography (SiO_2, PE /DCM, 1/1.5, V/V) to afford **BTFA5** (52 mg, 52.0%) as a red solid. ^{1}H NMR (600 MHz, Chloroform-*d*) δ 8.35 (s, 2H, ArH), 7.91 (d, *J* = 6.8 Hz, 2H, ArH), 7.69 (d, *J* = 6.7 Hz, 2H, ArH), 7.42-7.30 (m, 6H, ArH), 6.90-6.77 (m, 4H, ArH), 3.15 (s, 4H, $-CH_2-$), 2.93-2.84 (m, 4H, $-CH_2-$), 2.02 (s, 2H, $-CH_2-$), 1.73-1.66 (m, 2H, $-CH_2-$), 1.65-1.55 (m, 12H, $-CH_2-$), 1.52-1.24 (m, 52H, $-CH_2-$), 0.98-0.89 (m, 18H,

-CH_3), 0.86-0.80 (m, 6H, -CH_3). ^{13}C NMR (151 MHz, Chloroform-*d*) δ 194.88, 147.95, 142.95, 142.69, 140.87, 139.84, 137.62, 137.33, 136.26, 135.14, 134.59, 134.28, 134.08, 133.48, 131.21, 130.70, 128.23, 125.72, 124.03, 122.60, 121.81, 120.09, 116.55, 113.99, 113.92, 41.54, 39.12, 37.04, 34.11, 33.28, 32.29, 31.83, 31.76, 29.97, 29.71, 29.63, 29.52, 29.27, 28.93, 28.44, 26.48, 26.44, 25.36, 23.04, 22.63, 22.60, 14.22, 14.10, 14.05, 10.84, 1.02. MS: $[M+H]^+$ calcd for $C_{102}H_{117}N_4S_4^+$, 1526.8190; found, 1526.8037.

Synthesis of BTFA6

Compound **5** (120 mg, 79 μmol) and AgOTf (6 mg, 24 μmol) were dissolved in 5 mL of dry 1,2-dichloroethane under N_2. The mixture was heated to 70 °C and stirred for 72 h. After cooling to room temperature, it was concentrated using a rotary evaporator and purified through column chromatography (SiO_2, PE /DCM, 1/1.5, V/V) to afford **BTFA6** (90 mg, 75.7%) as an orange solid. ^{1}H NMR (400 MHz, Chloroform-*d*) δ 8.13-8.09 (m, 6H, ArH), 8.08 (d, *J* = 7.7 Hz, 2H, ArH), 7.86 (d, *J* = 7.2 Hz, 2H, ArH), 7.70 (dd, *J* = 7.8, 1.6 Hz, 2H, ArH), 7.04 (d, *J* = 3.5 Hz, 2H, ArH), 6.90 (d, *J* = 3.5 Hz, 2H, ArH), 2.89 (d, *J* = 6.6 Hz, 4H, -CH_2-), 2.71-2.55 (m, 4H, -CH_2-), 1.72-1.62 (m, 2H, -CH-), 1.55-1.34 (m, 16H, -CH-, -CH_2-), 1.25-0.93 (m, 62H, -CH-, -CH_2-), 0.82 (t, *J* = 7.1 Hz, 6H, -CH_3), 0.76 (t, *J* = 7.0 Hz, 6H, -CH_3).^{13}C NMR (151 MHz, Chloroform-*d*) δ 147.48, 140.13, 139.01, 136.53, 136.40, 135.34, 135.22, 135.16, 133.96, 133.90, 133.51, 133.13, 130.86, 130.61, 130.00, 129.40, 128.02, 125.54, 122.18, 121.71, 119.71, 119.33, 118.37, 117.39, 116.54, 113.67, 112.99, 41.41, 39.66, 38.64, 34.04, 33.81, 33.75, 32.33, 32.07, 31.97, 30.38, 30.06, 29.84, 29.48, 28.92, 26.81, 25.33, 23.09, 22.81, 22.73, 14.25, 14.23, 14.13, 10.80, 1.03. MS: $[M•2H_2O+H]^+$ calcd for $C_{102}H_{121}N_4O_2S_4^+$, 1562.8401; found, 1562.7908.

2. Supporting Figures

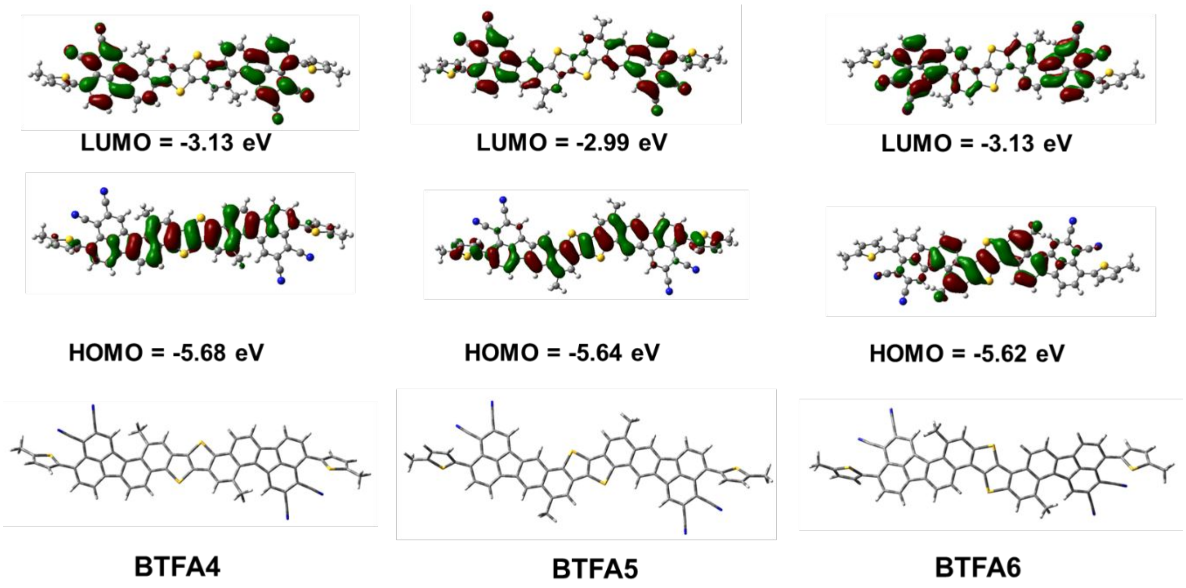

Figure S1. DFT-optimized molecular frontier orbitals and framework.

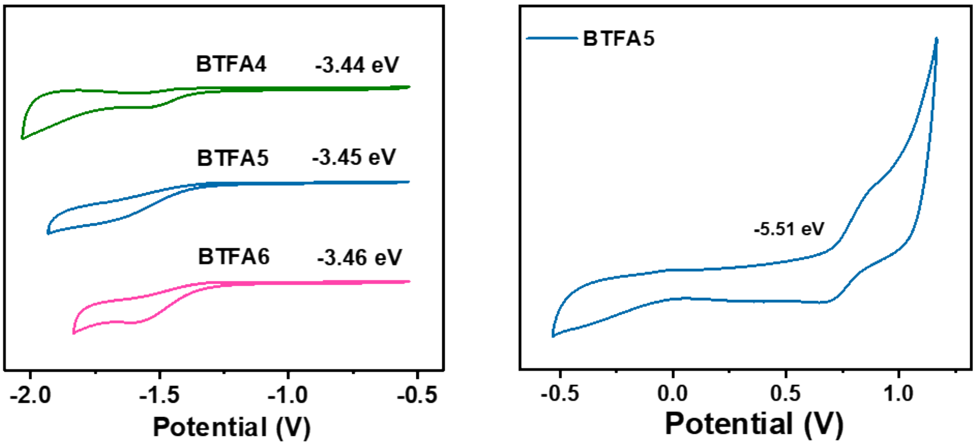

Figure S2. CV curves of **BTFA4-6** in the negative sweep and **BTFA5** in the positive sweep.

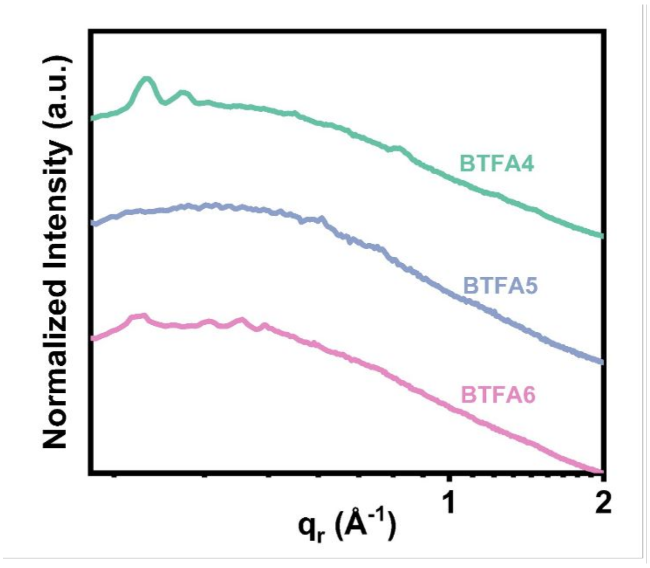

Figure S3. Corresponding 1D profiles of **BTFA4-6** extracted from the in-plane (IP) and out-of-plane (OOP) directions.

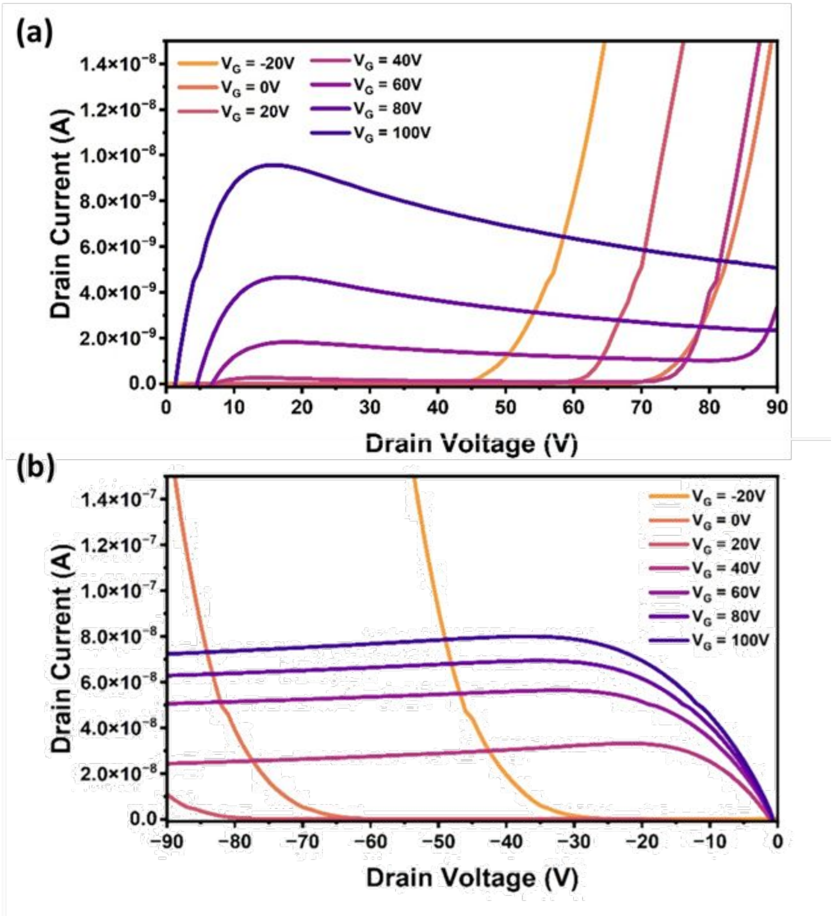

Figure S4. (a) N-type and (b) p-type output curves of the FET devices based on **BTFA5**.

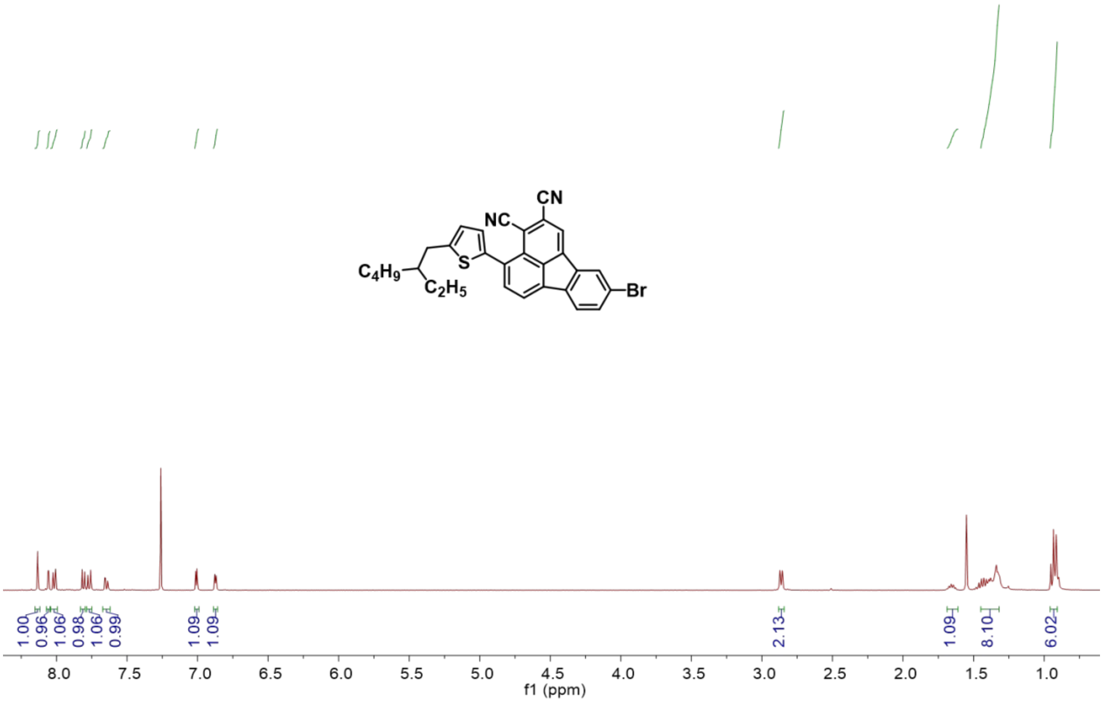

Figure S5. The ^{1}H NMR spectrum of compound **1b**, conducted in chloroform-*d*.

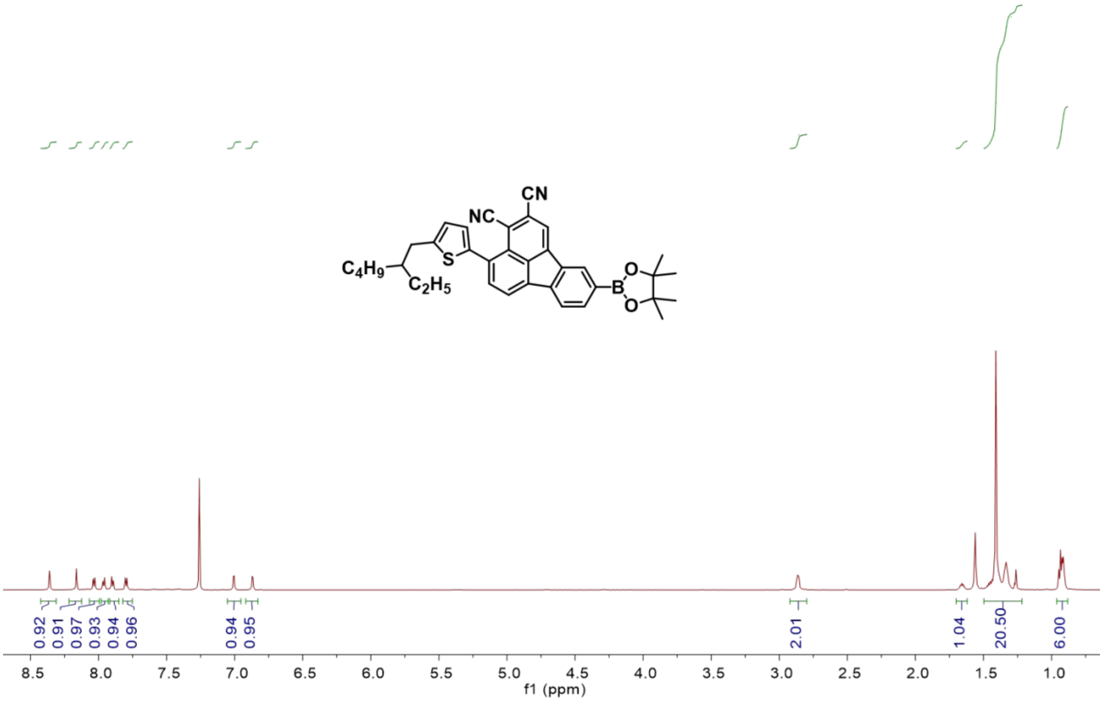

Figure S6. The ^{1}H NMR spectrum of compound **1**, conducted in chloroform-*d*.

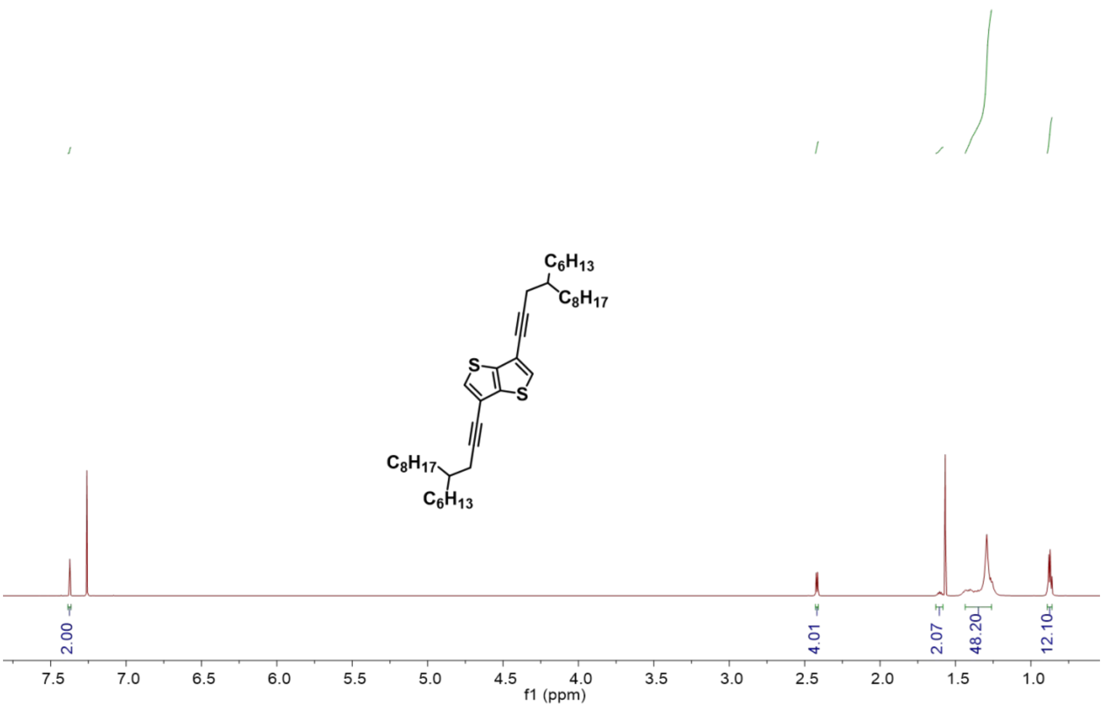

Figure S7. The ^{1}H NMR spectrum of compound **2b**, conducted in chloroform-*d*.

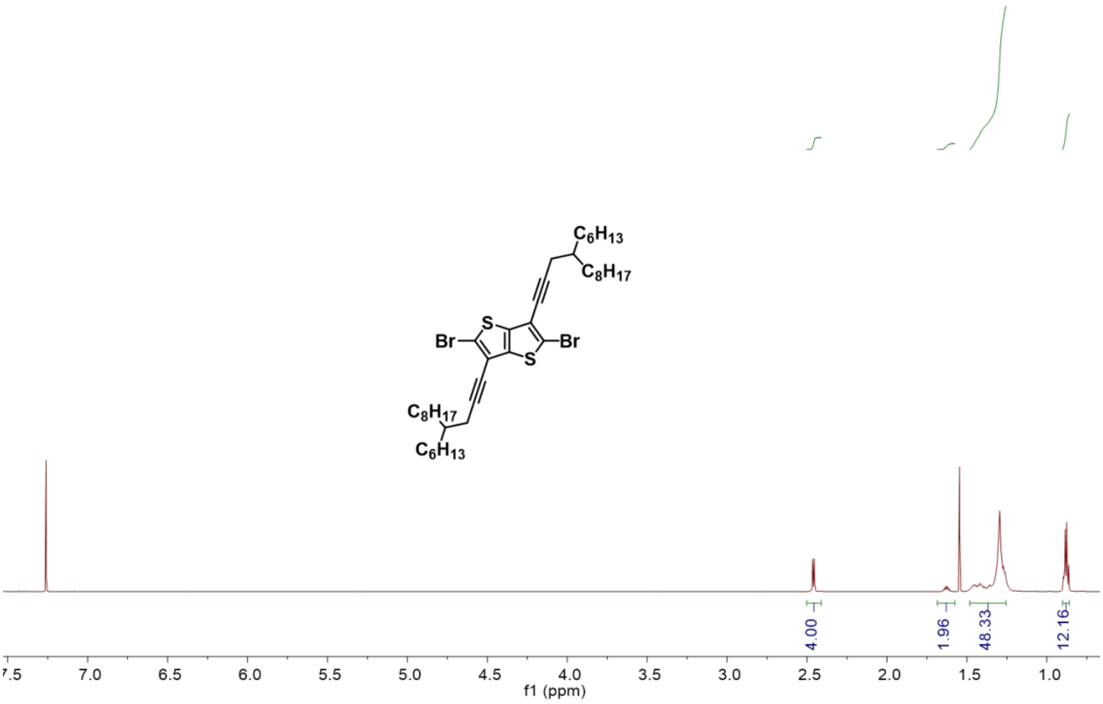

Figure S8. The ^{1}H NMR spectrum of **2**, conducted in chloroform-*d*.

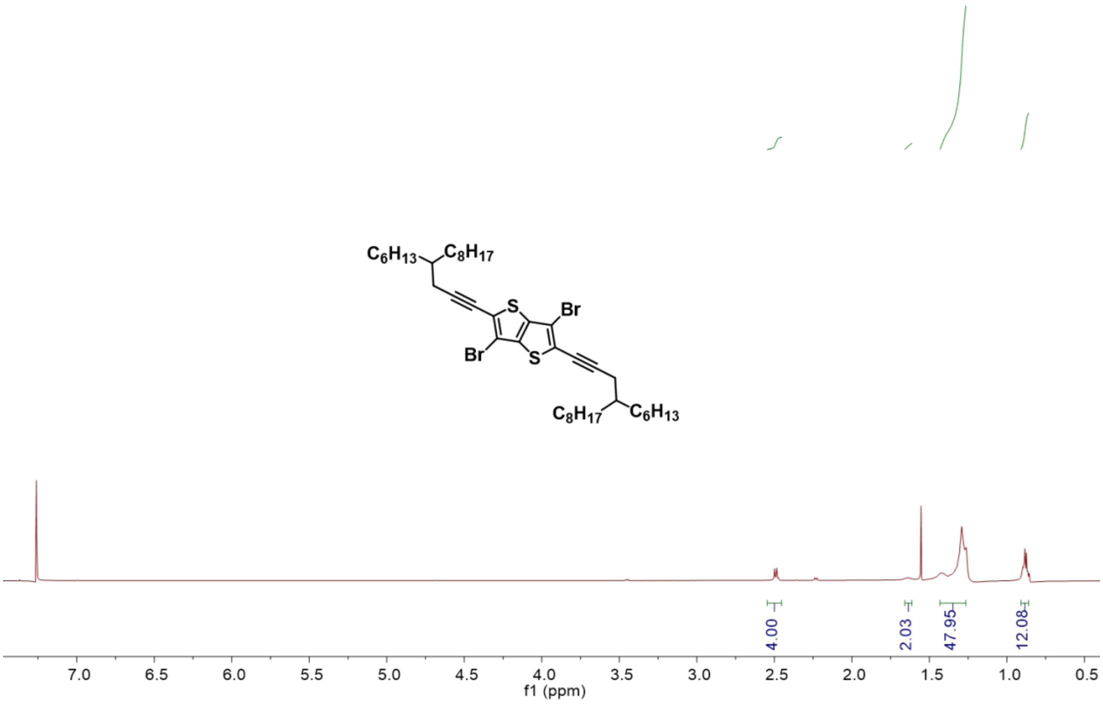

Figure S9. The ^{1}H NMR spectrum of **3**, conducted in chloroform-*d*.

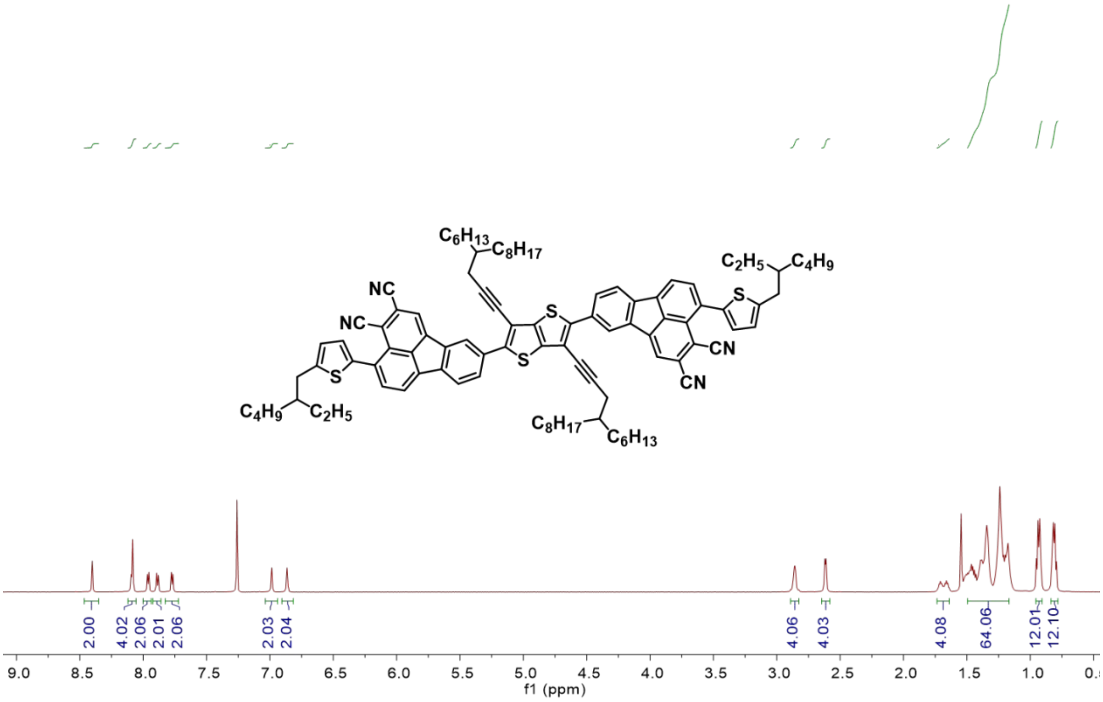

Figure S10. The ^{1}H NMR spectrum of **4**, conducted in chloroform-*d*.

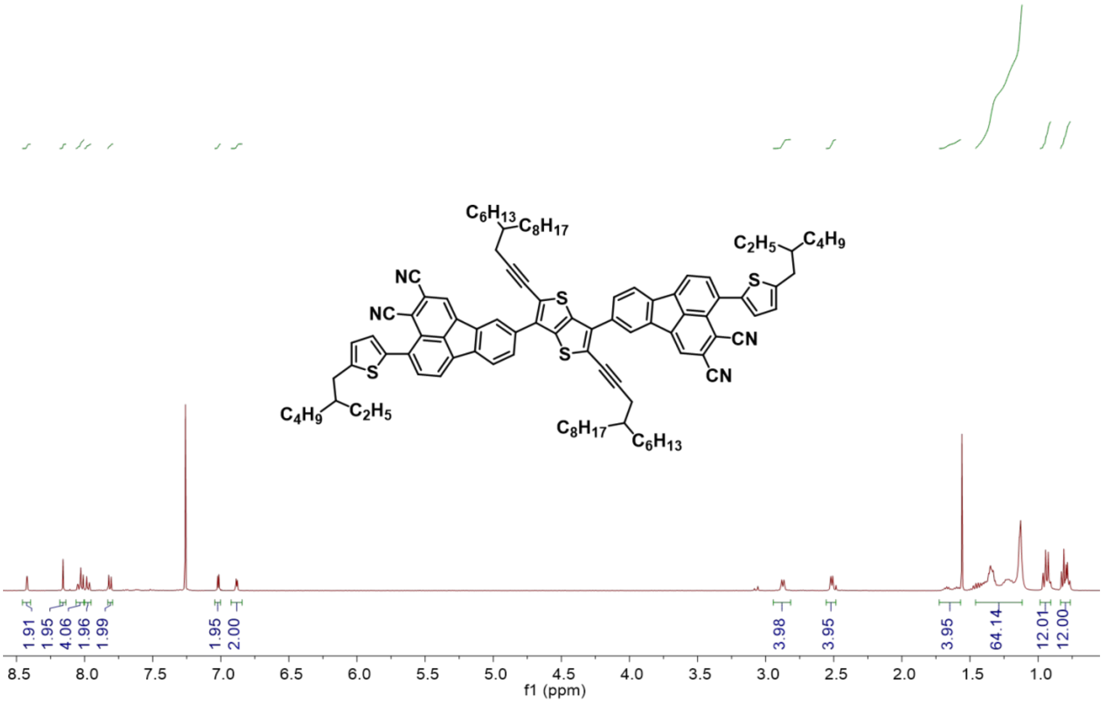

Figure S11. The ^{1}H NMR spectrum of **5**, conducted in chloroform-*d*.

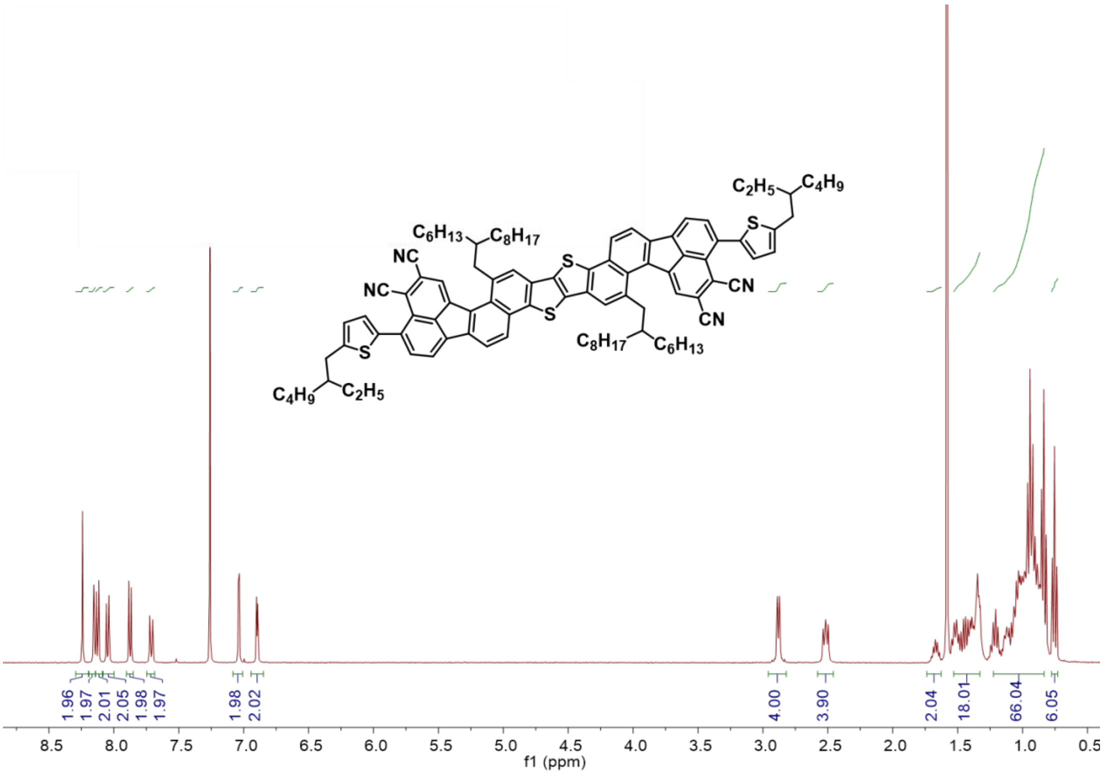

Figure S12. The ^{1}H NMR spectrum of **BTFA4**, conducted in chloroform-*d*.

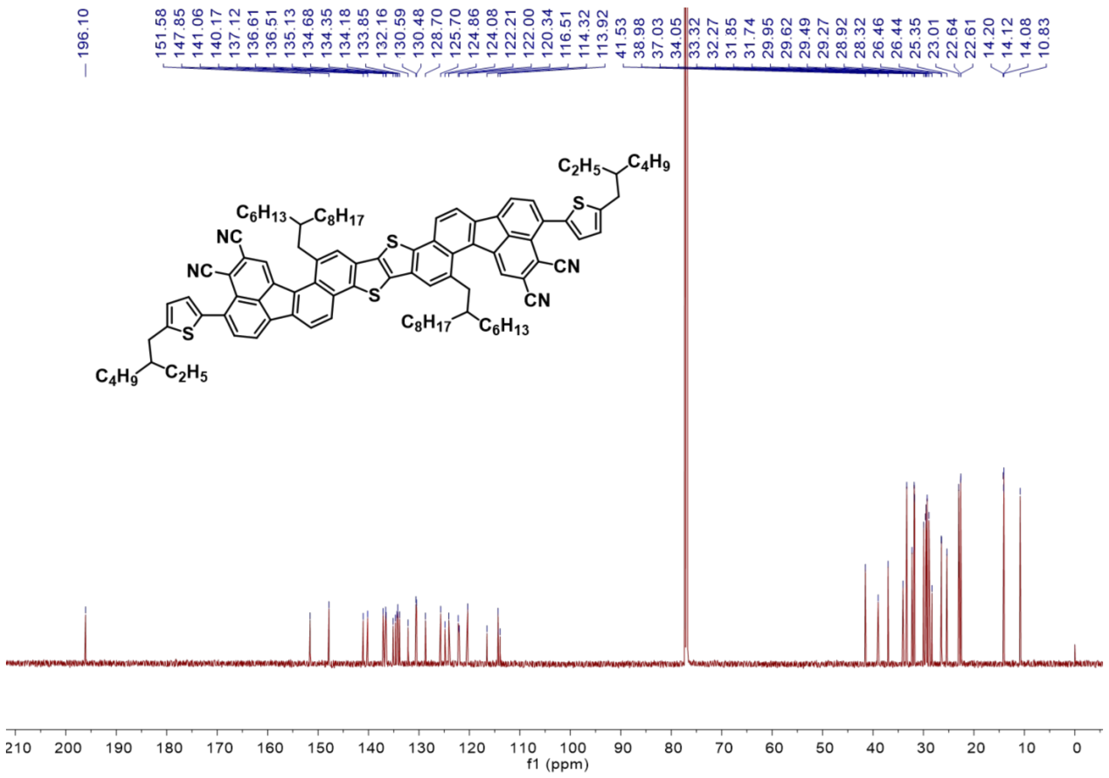

Figure S13. The ^{13}C NMR spectrum of **BTFA4**, conducted in chloroform-*d*.

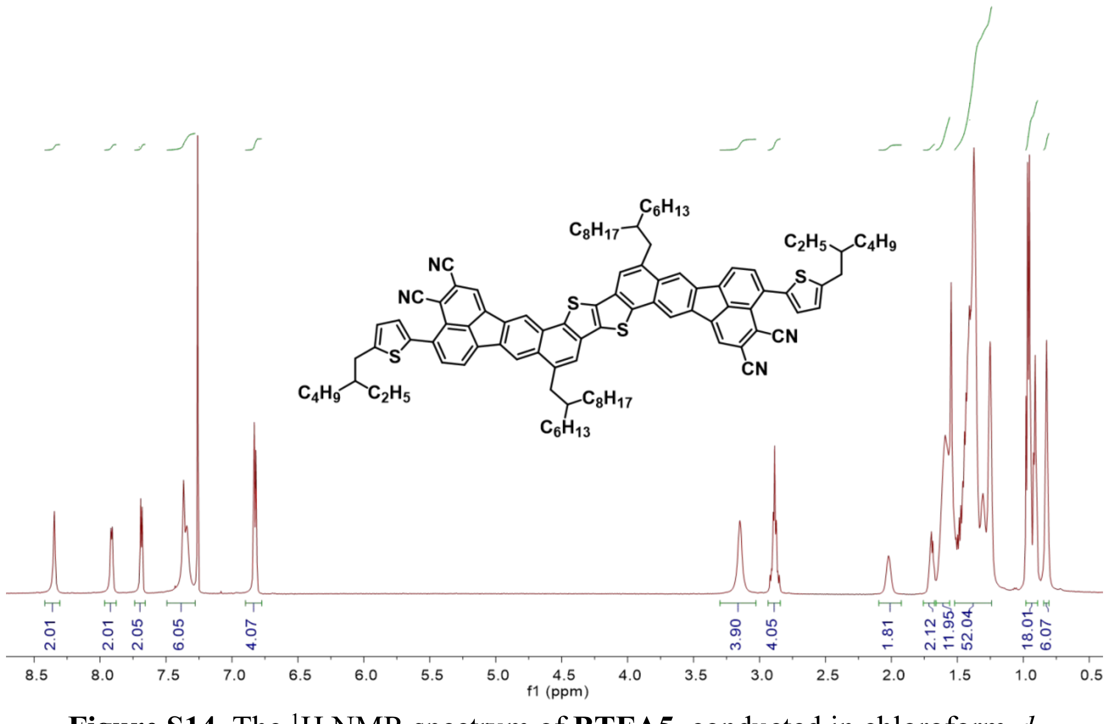

Figure S14. The ^{1}H NMR spectrum of **BTFA5**, conducted in chloroform-*d*.

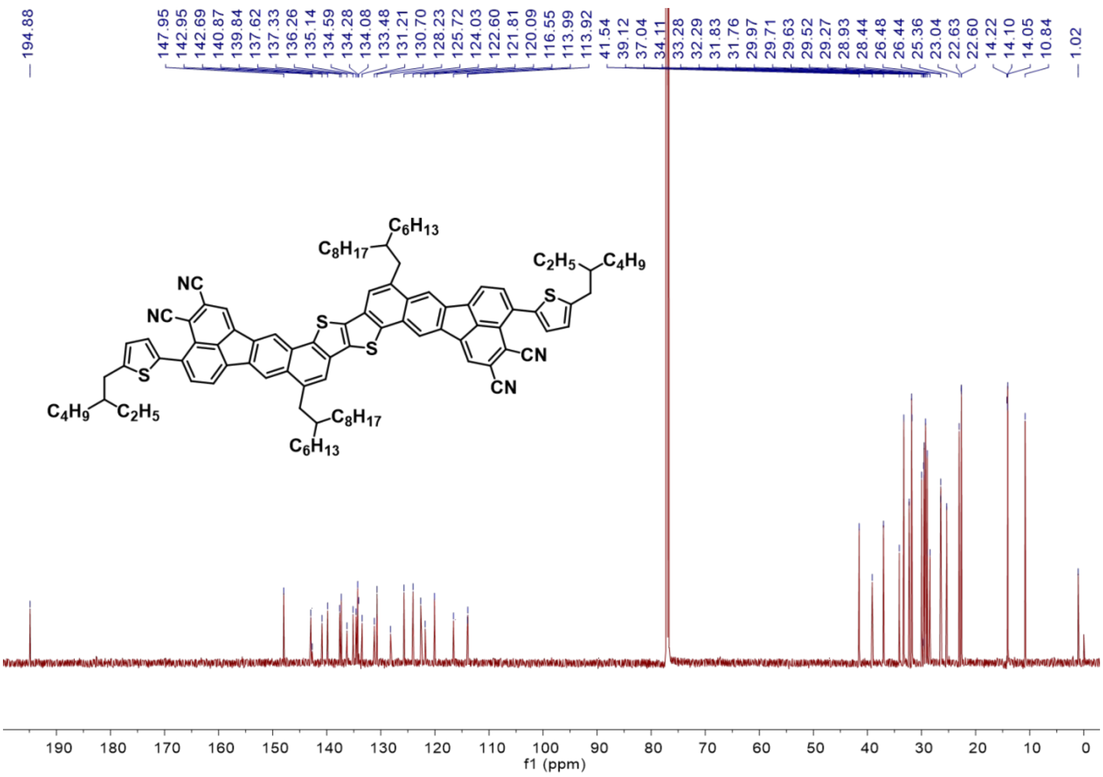

Figure S15. The ^{13}C NMR spectrum of **BTFA5**, conducted in chloroform-*d*.

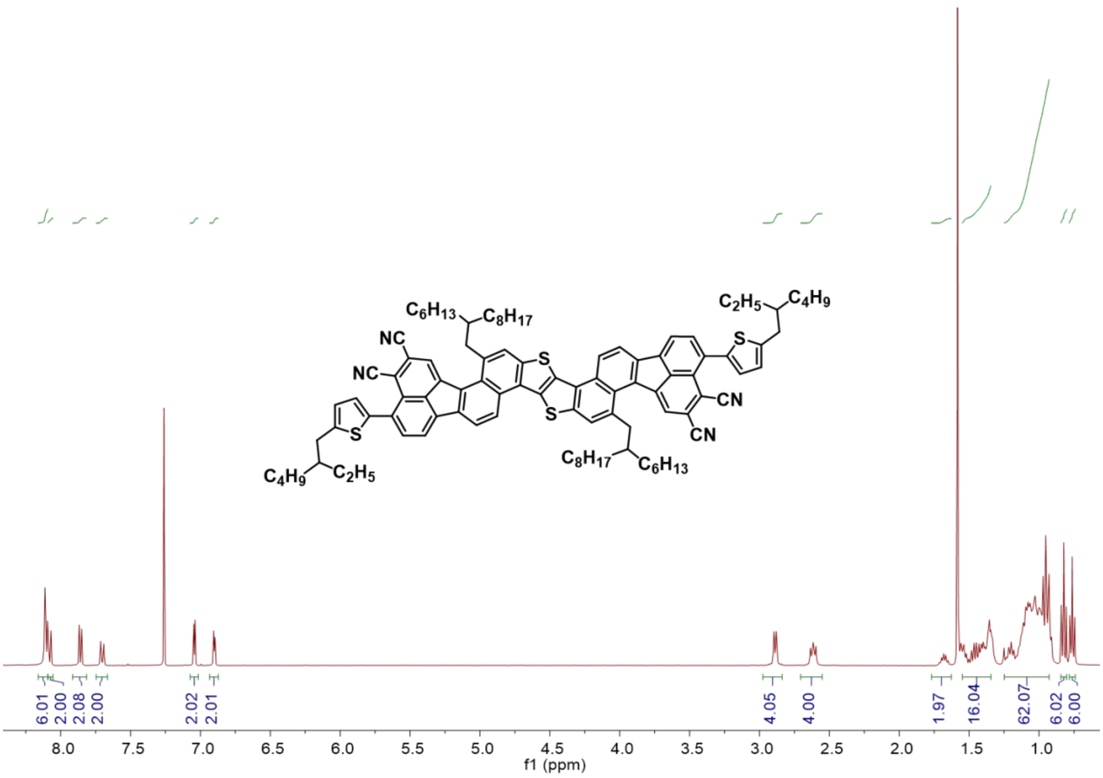

Figure S16. The ^{1}H NMR spectrum of **BTFA6**, conducted in chloroform-*d*.

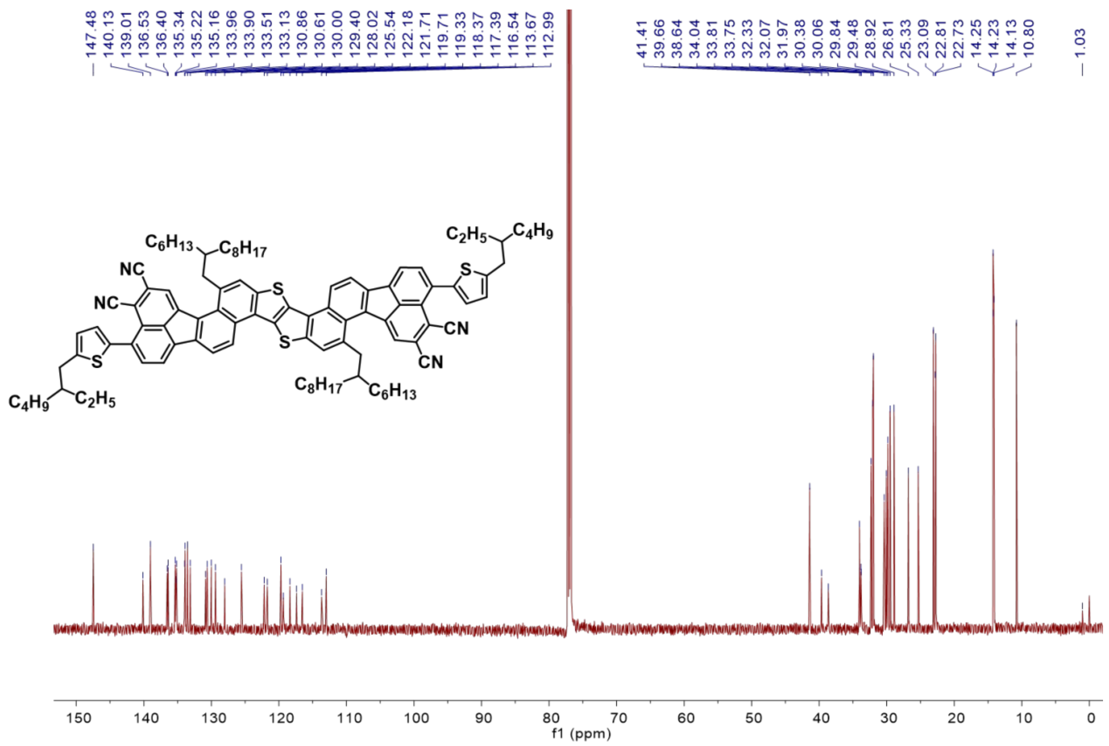

Figure S17. The ^{13}C NMR spectrum of **BTFA6**, conducted in chloroform-*d*.

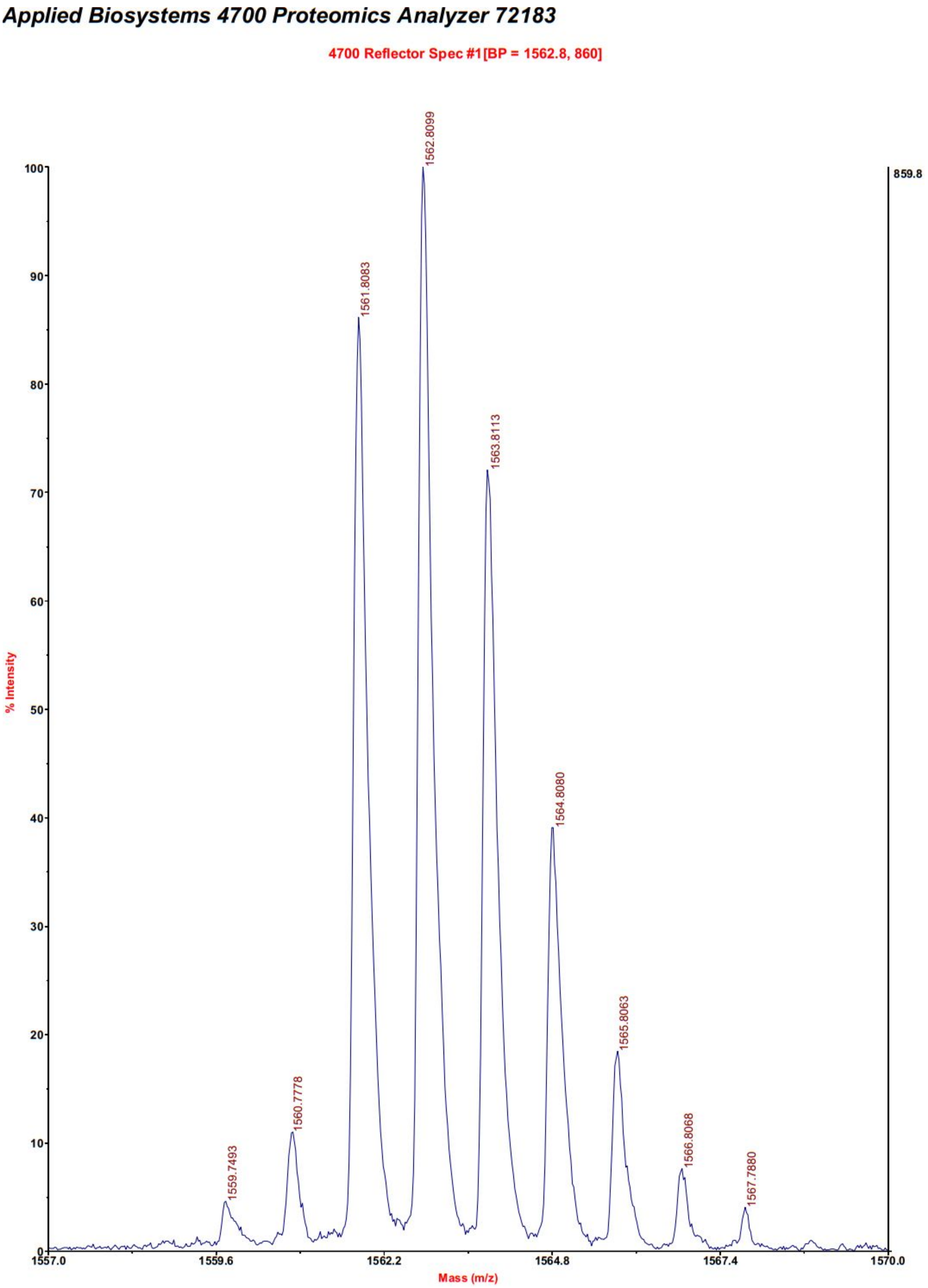

Figure S18. The Mass spectrum of **BTFA4**.

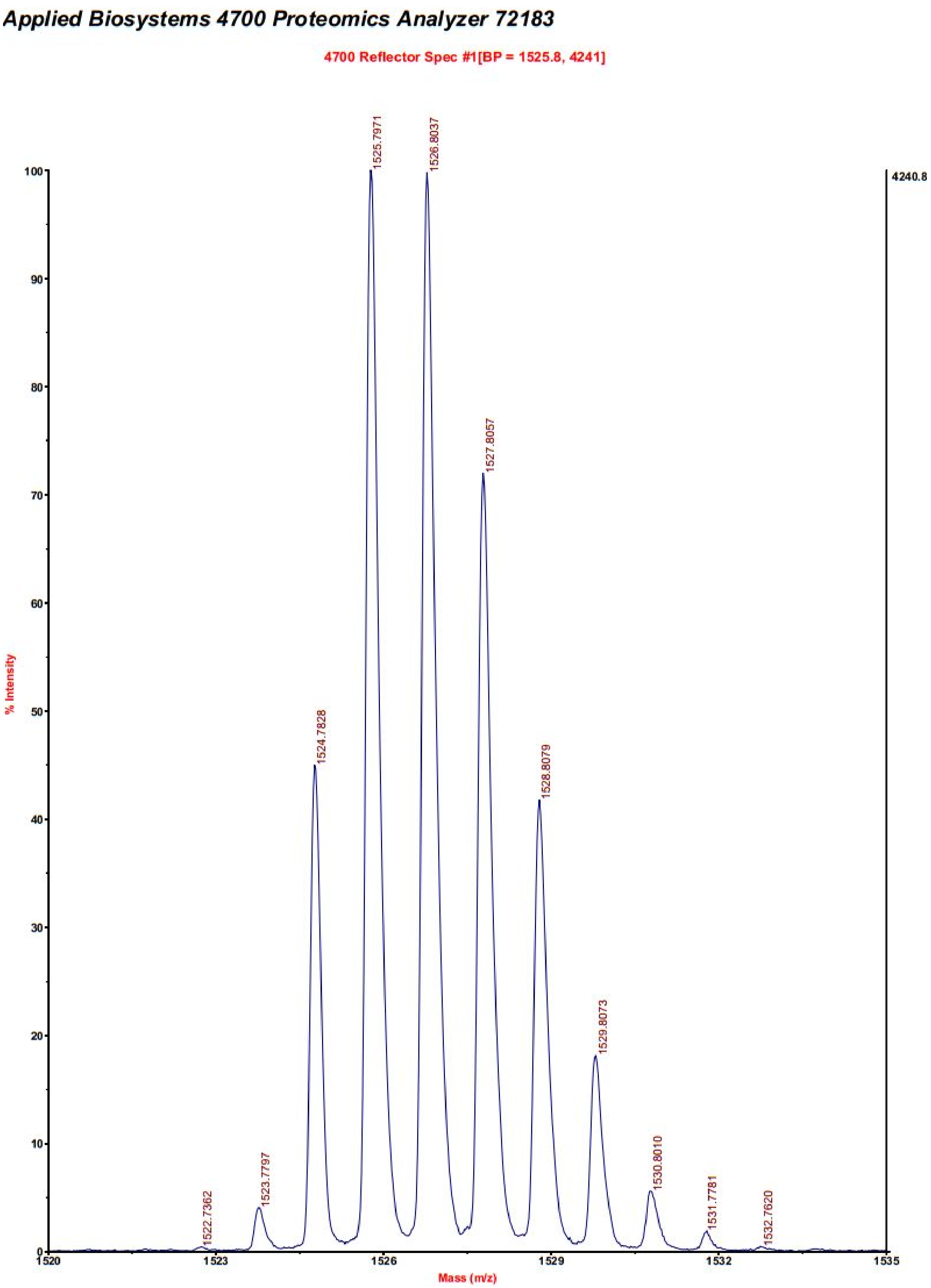

Figure S19. The Mass spectrum of **BTFA5**.

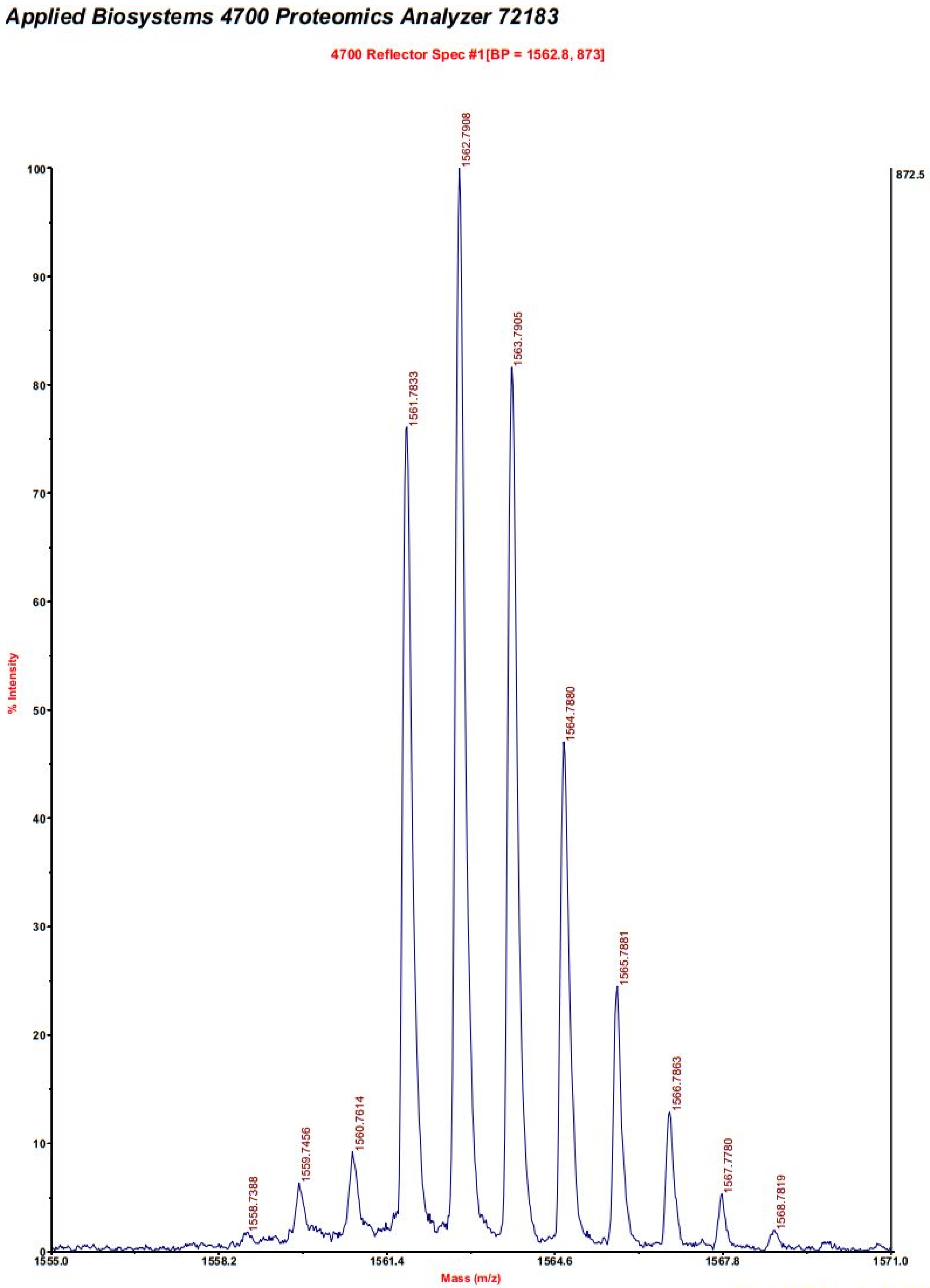

Figure S20. The Mass spectrum of **BTFA6**.

3. Reference

(1) Yu, X.; Li, Z.; Sun, X.; Zhong, C.; Zhu, Z.; Li, Z. a.; Jen, A. K. Y., Dopant-free dicyanofluoranthene-based hole transporting material with low cost enables efficient flexible perovskite solar cells. *Nano Energy* **2021,** *82*, 105701.

(2) Otep, S.; Ogita, K.; Yomogita, N.; Motai, K.; Wang, Y.; Tseng, Y.-C.; Chueh, C.-C.; Hayamizu, Y.; Matsumoto, H.; Ishikawa, K.; Mori, T.; Michinobu, T., Cross-linking of poly(arylenebutadiynylene)s and its effect on charge carrier mobilities in thin-film transistors. *Macromolecules* **2021,** *54* (9), 4351-4362.

(3) Galli, D.; Gasparini, N.; Forster, M.; Eckert, A.; Widling, C.; Killian, M. S.; Avgeropoulos, A.; Gregoriou, V. G.; Scherf, U.; Chochos, C. L.; Brabec, C. J.; Ameri, T., Suppressing the surface recombination and tuning the open-circuit voltage of polymer/fullerene solar cells by implementing an aggregative ternary compound. *ACS Appl. Mater. Interfaces* **2018,** *10* (34), 28803-28811.

(4) Shao, J.; Zhao, X.; Wang, L.; Tang, Q.; Li, W.; Yu, H.; Tian, H.; Zhang, X.; Geng, Y.; Wang, F., Synthesis and characterization of π-extended thienoacenes with up to 13 fused aromatic rings. *Tetrahedron Lett.* **2014,** *55* (41), 5663-5666.
